# The Δ133p53β isoform promotes an immunosuppressive environment leading to aggressive prostate cancer

**DOI:** 10.1038/s41419-019-1861-1

**Published:** 2019-08-20

**Authors:** Marina Kazantseva, Sunali Mehta, Ramona A. Eiholzer, Gregory Gimenez, Sara Bowie, Hamish Campbell, Ashley L. Reily-Bell, Imogen Roth, Sankalita Ray, Catherine J. Drummond, Glen Reid, Sebastien M. Joruiz, Anna Wiles, Helen R. Morrin, Karen L. Reader, Noelyn A. Hung, Margaret A. Baird, Tania L. Slatter, Antony W. Braithwaite

**Affiliations:** 1 Department of Pathology, Dunedin School of Medicine, University of Otago, Dunedin, New Zealand; 2 Maurice Wilkins Centre for Molecular Biodiscovery, Auckland, New Zealand; 3 Children’s Medical Research Institute, University of Sydney, Camperdown, NSW 2145 Australia; 4 Jacqui Wood Cancer Centre, Division of Cancer Research, University of Dundee, Dundee, UK; 5 Department of Pathology, University of Otago, Christchurch, New Zealand; 6 Department of Anatomy, School of Biomedical Sciences, University of Otago, Dunedin, New Zealand

**Keywords:** Cancer microenvironment, Tumour-suppressor proteins

## Abstract

Prostate cancer is the second most common cancer in men, for which there are no reliable biomarkers or targeted therapies. Here we demonstrate that elevated levels of *Δ133TP53β* isoform characterize prostate cancers with immune cell infiltration, particularly T cells and CD163+ macrophages. These cancers are associated with shorter progression-free survival, Gleason scores ≥ 7, and an immunosuppressive environment defined by a higher proportion of PD-1, PD-L1 and colony-stimulating factor 1 receptor (CSF1R) positive cells. Consistent with this, RNA-seq of tumours showed enrichment for pathways associated with immune signalling and cell migration. We further show a role for hypoxia and wild-type p53 in upregulating *Δ133TP53* levels. Finally, AUC analysis showed that *Δ133TP53β* expression level alone predicted aggressive disease with 88% accuracy. Our data identify *Δ133TP53β* as a highly accurate prognostic factor for aggressive prostate cancer.

## Introduction

Prostate cancer is the second most common cancer among men worldwide with >250,000 deaths each year^1^. The multifactorial aetiology of prostate cancer is linked to age, genomic alterations, diet and inflammation^2–4^. Mutations in the tumour suppressor p53 (*TP53*) gene have been implicated in prostate cancer progression^5^; however, they do not reliably predict aggressive disease^5^.

The *TP53* gene encodes 12 isoforms through the use of alternative promoters, translation start sites and RNA splicing^6,7^. In addition to full-length p53 (FLp53) isoforms, there are three sets of isoforms (Δ40p53, Δ133p53, and Δ160p53) that lack the N terminus and are alternatively spliced at the C terminus resulting in three variants: *α*, *β*, and *γ*^6,7^. Multiple pro-tumorigenic functions have been attributed to Δ133p53 including promoting cell cycle progression^8–11^, anti-apoptotic activity^12^, angiogenesis^13^, migration^14,15^, increased DNA repair^16^, reduced chemosensitivity^17,18^ and increased telomerase activity^11^.

Δ133p53 may also contribute to cancer by promoting inflammation. Mice constitutively expressing a ‘mimic’ of ∆133p53 (∆122p53) had elevated pro-inflammatory serum cytokines^9,14,19^ and ∆122p53 expressing mouse embryonic fibroblasts had elevated levels of IL-6 and several chemokines^14,18^. In peripheral blood mononuclear cells from ∆122p53 mice, and gastric carcinoma cells transfected with ∆133p53α, there was increased NF-κB activity^20,21^, suggesting that ∆133p53 isoforms may drive this canonical inflammatory signalling pathway.

Inflammatory cells within prostate cancers can promote angiogenesis and epithelial mesenchymal transition leading to metastatic disease^3^ and an immunosuppressive milieu has been shown to correlate with advanced disease and therapeutic inefficacy^22,23^. Additionally, we have shown that brain tumours with a high content of tumour-associated macrophages (TAM) had elevated *Δ133TP53β* mRNA levels^18^, suggesting that ∆133p53 promotes immune cell migration. To expand this observation, in this paper we investigated a link between Δ133p53 isoforms, immune cell infiltration and tumour progression in prostate cancers.

We report here that elevated *Δ133TP53β* is a key feature of prostate cancers with an increased proliferative index, high immune cell infiltrate, and an immunosuppressive phenotype. We also show that *Δ133TP53β* mRNA levels can predict which patients are likely to develop advanced disease.

## Results

### *Δ133TP53β* expression is elevated in a subset of prostate cancers

An association between Δ133p53 and inflammation has not been investigated in prostate cancer. Here we quantified transcript levels of full-length p53 (*FLTP53*) and all *TP53* isoforms using RT–qPCR in 122 prostate cancers from two separate cohorts of patients (*n* = 43; *n* = 79) and 3 non-neoplastic prostate samples.

**Fig. 1:**
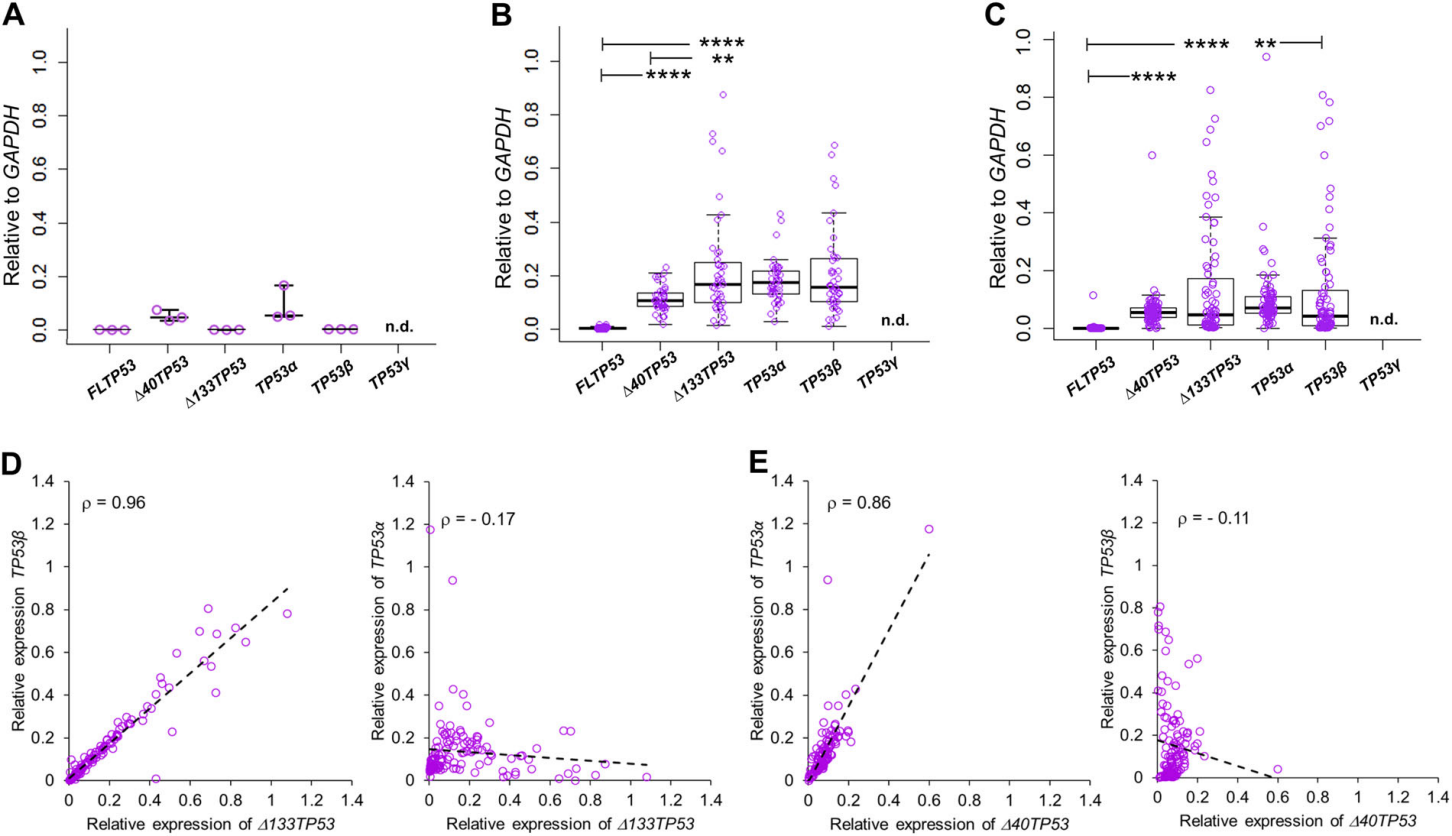
The p53 isoform, *Δ133TP53β*, is upregulated in prostate cancers. Box and whisker plots show relative *TP53* transcript levels in **a**. Non-neoplastic prostate tissues (*n* = 3) and in 2 prostate cancer cohorts: **b** cohort 1 (*n* = 43) and **c**. cohort 2 (*n* = 79). **a**–**c** Symbols show individual cancers; the horizontal line in the box represents median values and the outlines represent the 25th–75th percentile, whiskers show the 10–90% CI. Significant differences were determined by Mann–Whitney *U* test, *p < 0.05, **p < 0.01, ***p < 0.005, . n.d. not detected. Scatter plots show the correlation of relative abundance of **d**. ∆*133TP53* vs *TP53β* transcripts *p* < 0.0001 (left panel) and *∆133TP53* vs *TP53α* transcripts *p* = 0.24 (right panel). **e** ∆*40TP53* vs *TP53α* transcripts *p* < 0.0001 (left panel), and ∆*40TP53* vs *TP53β* transcripts *p* = 0.21 (right panel), from both prostate cancer cohorts (*n* = 122). Spearman *ρ* values are shown

Overall, the median expression levels of all isoform transcripts were found to be higher than for non-neoplastic tissue (Fig. 1a–c) with the exception of *FLTP53*. *Δ133TP53* had a higher expression range compared to *Δ40TP53* in both patient cohorts (Fig. 1b, c) and had a higher median expression than *Δ40TP53* in cohort 1. The *Δ133TP53* variant has a strong positive correlation with the β isoform (*TP53β*) (*ρ* = 0.96, *p* < 0.0001; Fig. 1d) but not with the α isoform (*TP53α*, Fig. 1d), suggesting that *Δ133TP53β* is the predominant *Δ133TP53* isoform. On the other hand, there was a high correlation between *Δ40TP53* and *TP53α* expression (*ρ* = 0.86; *p* < 0.0001) but not with *TP53β* (Fig. 1e); suggesting that most of the *TP53α* transcript is associated with *Δ40TP53α*. No *TP53γ* isoform was detected in either non-neoplastic or cancer tissues (Fig. 1a–c). These data suggest *Δ133TP53β* and *Δ40TP53α* mRNAs are increased in subsets of prostate cancers.

One explanation for the elevated expression could be due to *TP53* mutations affecting mRNA stability. We therefore sequenced the *TP53* gene from 39/122 cancers. Overall, *TP53* mutations were found in 18% (7/39) of prostate cancers making it unlikely that this accounts for increased isoform expression.

### Elevated expression of *Δ133TP53β* mRNA in prostate cancers is associated with inflammation

**Fig. 2:**
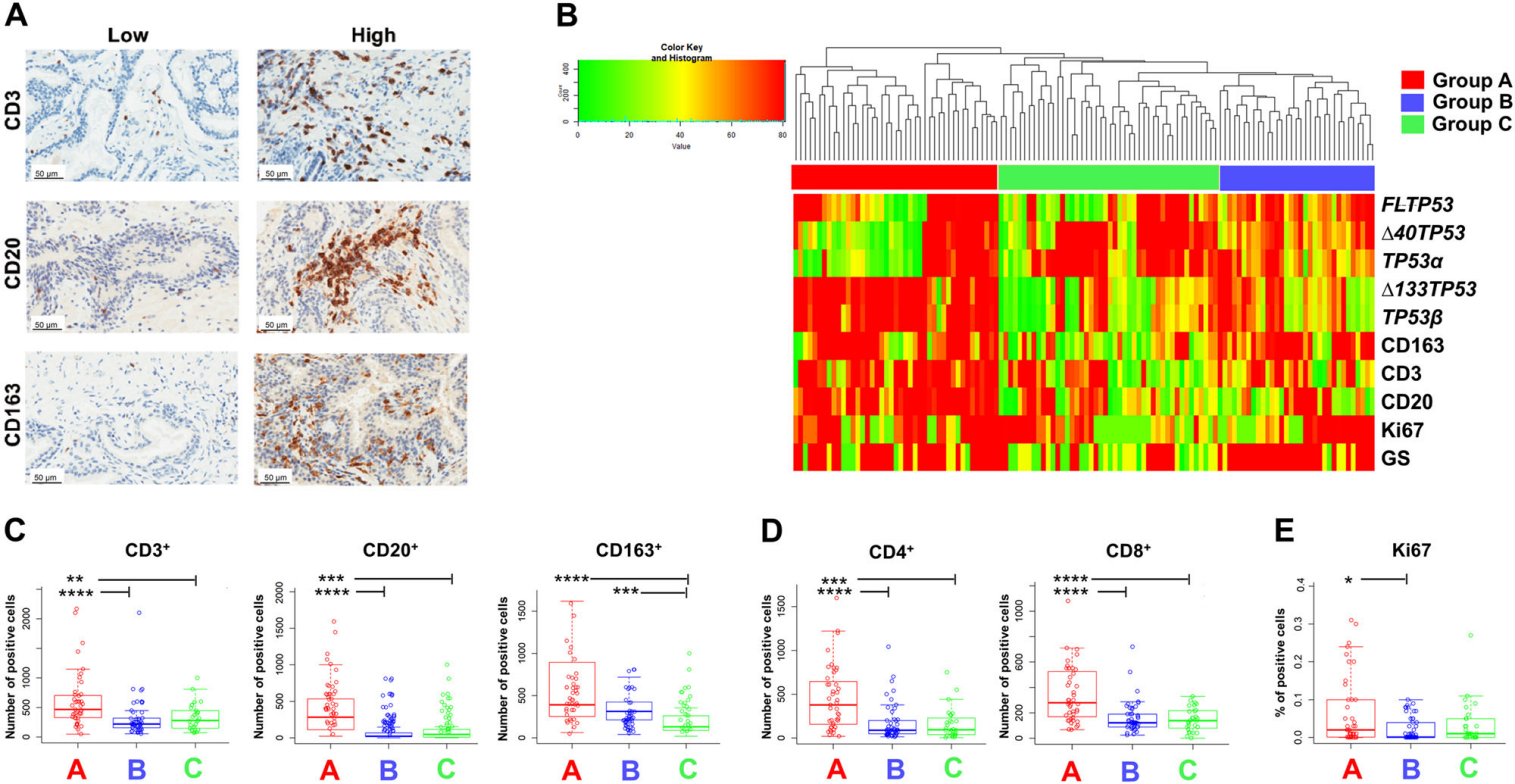
Prostate cancers with elevated *Δ133TP53β* show high immune cell infiltration and increased proliferation. **a** Representative examples of prostate cancer sections with low- or high number of cells staining for CD3, CD20 and CD163 using immunohistochemistry. Magnification, ×200. **b** Unsupervised rank hierarchical clustering of 122 prostate cancers clustered by ranked mRNA expression of *FLTP53*, *Δ40TP53*, *TP53α*, *Δ133TP53*, *TP53β*, immune marker cell count CD163, CD3, CD20, proliferation marker Ki67 and the Gleason score (GS), identified three cancer subgroups designated Groups A (red), B (blue) and C (green). Box and whisker plots show **c**. the number of CD3+, CD20+ and CD163+ immune cells, **d** the number of CD4+ and CD8+ T cells in the three prostate groups and **e** the percentage of Ki67+ malignant cells as a measure of proliferation. Symbols show individual samples, box (median ± 25th–75th percentile), and whiskers show the 10–90% CI. *p < 0.05, **p < 0.01, ***p < 0.001, ****p < 0.0001 as determined by Kruskal–Wallis and Dunn’s post hoc test or by unpaired one-tailed *t*-test

As the Δ122p53 mice (expressing a mimic of the Δ133p53 isoform) provoke a pro-inflammatory environment, including secreting several cytokines and chemokines^9^ and brain cancers with high levels of *Δ133TP53β* had many infiltrating immune cells^18^, we quantitated the number of T-cells (CD3), B-cells (CD20) and macrophages (CD163) in the prostate cancer cohorts using immunohistochemistry (IHC). Results showed there was considerable immune cell infiltration but the extent of infiltration was variable, examples of which are shown in Fig. 2a. To determine whether there was any association between expression of *TP53* variants, immune cell infiltration and cancer aggressiveness, unsupervised rank ordered hierarchical clustering of *FLTP53*, *Δ40TP53, Δ133TP53*, *TP53α*, *TP53β* mRNA levels, immune cell content, the proliferation marker Ki67, and the Gleason score (GS) was performed. Clustering analysis (Fig. 2b) generated three groups of patients designated Group A (*n* = 43; 35%), Group B (*n* = 50; 41%) and Group C (*n* = 29; 24%) with Group A being characterized by high *Δ133TP53* and *TP53β* compared to Groups B and C (Fig. 2b). Group A cancers had significantly higher numbers of infiltrating CD3+T-cells, CD4+T-cells, CD8+T-cells and CD20+B-cells compared to Groups B and C and Groups A and B cancers had significantly higher numbers of infiltrating CD163+ macrophages compared to Group C (Fig. 2c, d). Group A also had a higher number of Ki67 positive cells compared to Group B (Fig. 2e). Thus, as was found for brain cancers^18^, prostate cancers with increased *Δ133TP53β* levels are associated with increased immune cell infiltration. Normal associated tissue was available for 30 prostate cancer samples. We therefore compared isoform levels in the cancers to the normal adjacent tissue from the same individual (Supplementary Fig. S1). In general all isoforms were elevated in Group A cancers and *FLTP53* and *Δ40TP53* were elevated in Groups B and C. Thus, elevated isoform mRNA levels tend to be a feature of prostate cancers.

### *Δ133TP53β* is expressed in prostate cancer cells

**Fig. 3:**
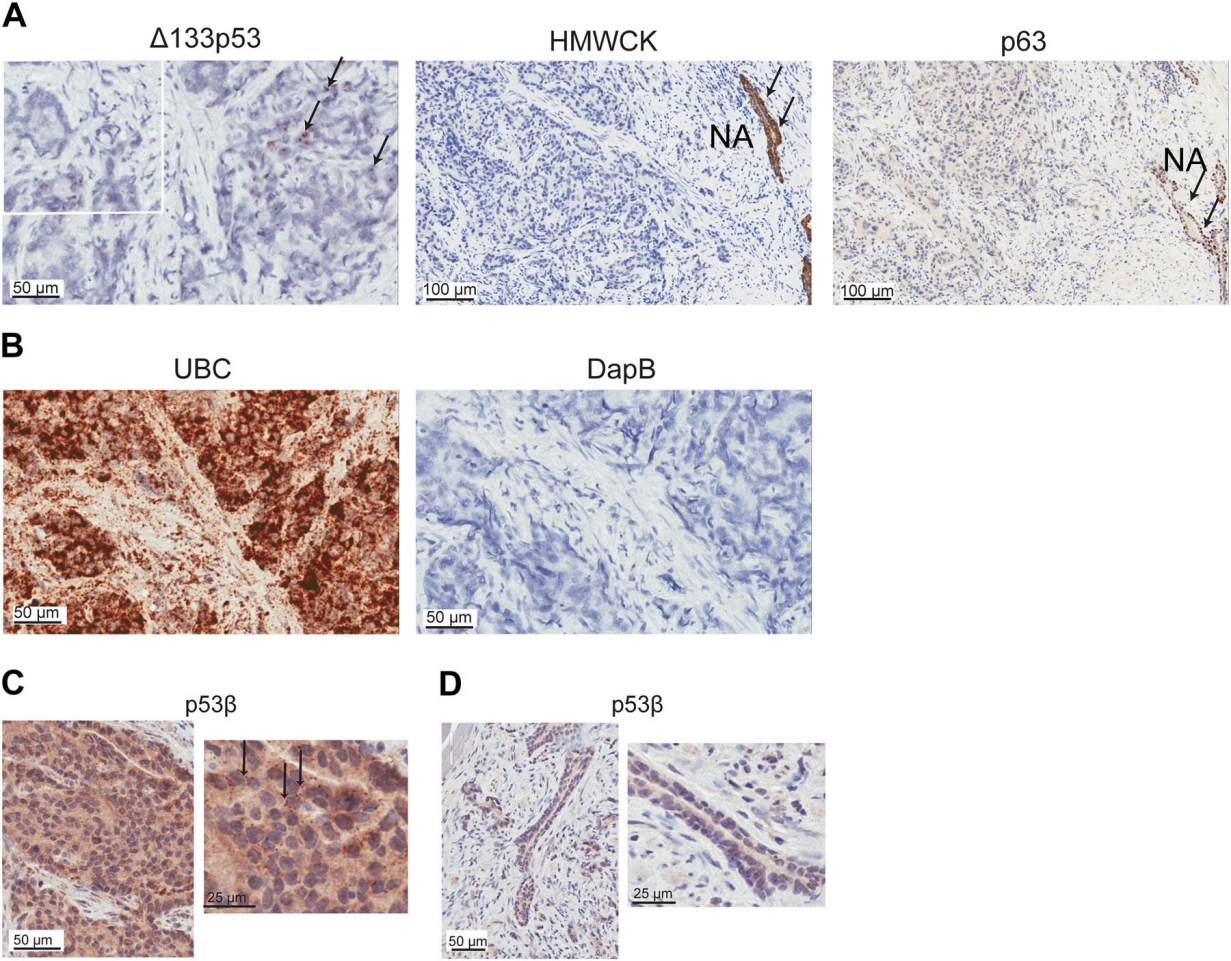
Δ133*TP53β* is expressed in cancer cells. **a** In situ hybridization using RNAscope detected *Δ133TP53* (black arrows) in FFPE prostate cancer tissues (left panel). Immunohistochemical staining for high molecular weight cytokeratin (HMWCK) and p63 (middle and right panels, respectively) to identify loss of HMWCK and p63 in prostate cancer. NA normal associated tissue (black arrows). **b** Left panel, probes to *ubiquitin C* (*UBC*) as a positive control for RNA quality and right panel, probes to the bacterial gene *DapB* as a negative control. Nuclei were counterstained with hematoxylin. **c** Immunohistochemistry using the KJC8 antibody to detect p53β (black arrows) in FFPE prostate cancer tissues. **d** Absence of p53β staining in normal associated prostate epithelium. Nuclei were counterstained with hematoxylin

We next asked whether the elevated *Δ133TP53β* levels in the cancer tissue samples were from the cancer cells or from the immune (or other) cells. This was done by a combination of RNAscope^18^ and IHC. The *Δ133TP53* RNAscope assay was optimized using the MCF7 breast cancer cell line that expresses *Δ133TP53* and compared with the *TP53* null Saos-2 osteosarcoma cell line (Supplementary Fig. S2)^6,24,25^. The *Δ133TP53* probe, a positive control probe to *ubiquitin C* (UBC) to check RNA quality, and a *DapB* negative control probe were hybridized to 15 tumours with the highest *Δ133TP53β* expression. The *Δ133TP53* probe showed positive staining (small brown dots) in some cells in all cancers (Fig. 3a, top left hand panel and inset). The isoform expression was in tissue regions that did not stain with epithelial cell markers p63 and high molecular weight cytokeratin (HMWCK), which was predominantly in the non-malignant epithelial tissue adjacent to the cancer (NA) (Fig. 3a, right hand panels; Supplementary Fig. S3), demonstrating that *Δ133TP53* is expressed in malignant cells (Fig. 3a). *Δ133TP53β* was not detected in lymphocyte aggregates (data not shown). Five cancer samples had scattered positive cells in the stroma, but overall few stromal cells were positive. As controls, strong diffuse staining with the *UBC* probe (positive control) was observed and no detectable staining was evident with the *DapB* (negative control, Fig. 3b).

To confirm that increased *Δ133TP53β* mRNA resulted in increased protein, IHC using the KJC8 antibody specific for p53β containing isoforms^6^ was done on the same 15 prostate cancers. The antibody was again optimized (Supplementary Fig. S4). Results from the cancer tissue analysis showed both diffuse cytoplasmic staining and stronger punctate staining in a subset of cancers (examples shown in Fig. 3c and Supplementary Fig. S5). Adjacent non-malignant prostate epithelial cells showed weak cytoplasmic staining without the stronger punctate staining (Fig. 3d).

### Elevated *Δ133TP53β* mRNA defines high-risk prostate cancer patients

**Fig. 4:**
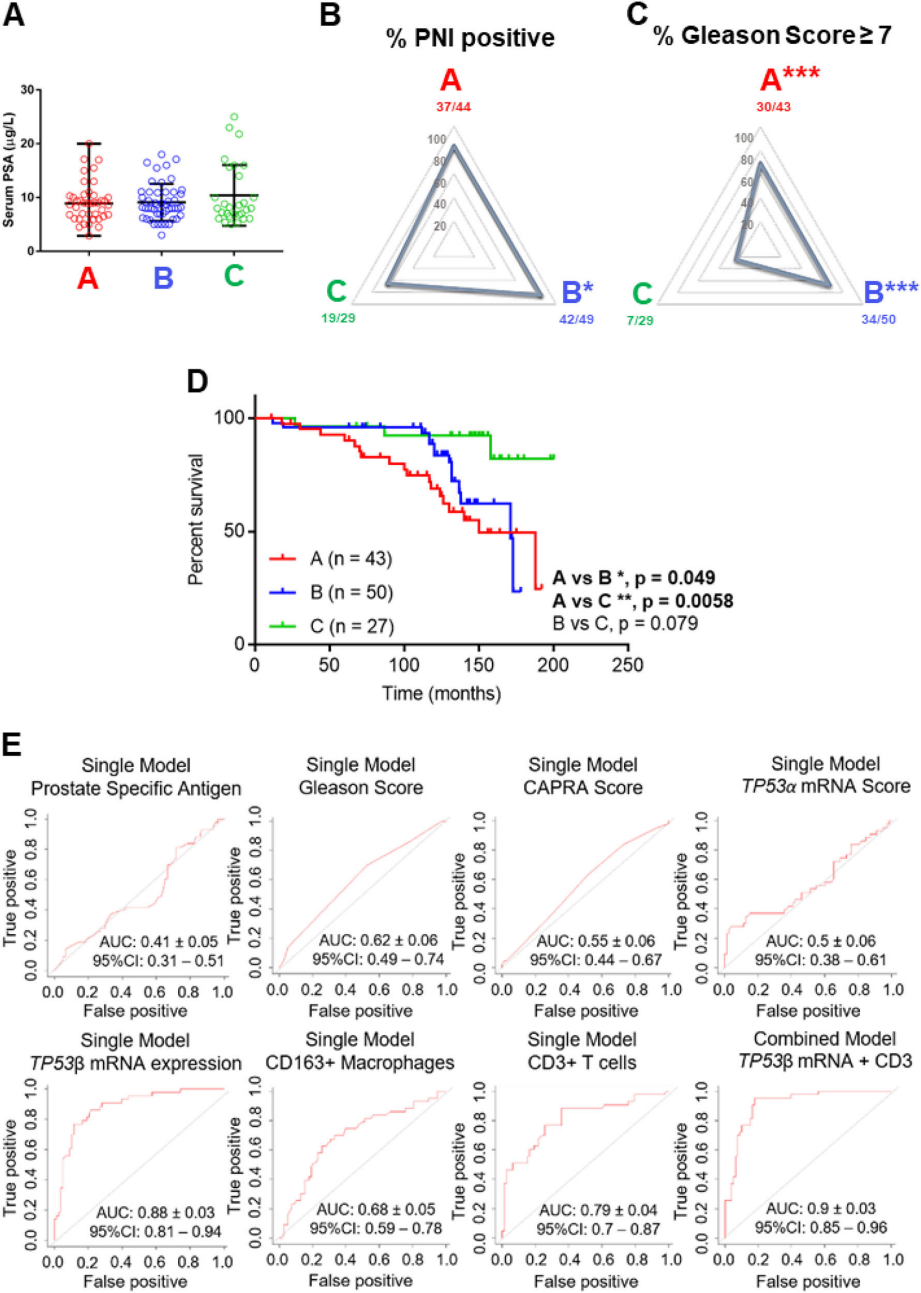
Elevated *Δ133TP53β* predicts prostate cancer patients at risk for developing aggressive cancer. Groups A, B and C prostate cancers were evaluated based on clinico-pathological criteria. **a** Total serum prostate-specific antigen (PSA) concentrations. Symbols show individual samples; horizontal lines represent median values and vertical lines represent the range. **b** Radar plots showing the frequency of cancers with perineural invasion (PNI). **c** Radar plots showing the frequency of cancers with Gleason Scores ≥7. Statistical significance was determined by chi-square test compared to Group C cancers. **d** Kaplan–Meier plots of progression-free survival of each subgroup of prostate cancers. Statistical significance was evaluated using log-rank test. **e** Receiver-operating characteristic (ROC) curve illustrating the 10-fold cross-validated area under the curve (AUC) ± SEM for predicting the probability of high-risk patients using either univariate or multivariate analyses. Top panel (left to right) prostate-specific antigen (PSA), Gleason Score, CAPRA score and *TP53α* mRNA expression. Bottom panel (left to right) *TP53β* mRNA expression, CD3+ T cells, CD163+ macrophages and the combined model of *TP53*β mRNA expression with CD3 immune cell content

We next tested the relationship between *Δ133TP53β* expression with clinical markers and progression-free patient survival (PFS). There were no differences in total PSA levels between the groups (median levels in Group A = 8.9 μg/L; B = 8.2 μg/L; C = 8.0 μg/L; Fig. 4a). However, Group B had more perineural invasion compared to Group C cancers (*p* = 0.036, chi-square test; Fig. 4b). Prostate cancers characterized by high *∆133TP53β* mRNA (Group A) or high *∆40TP53α* (Group B) cancers had a higher Gleason score ≥7 than Group C (Group A vs C: OR = 7.25, 95% CI: 2.48–19.85; *p* = 0.0001; and Group B vs C: OR = 6.68, 95% CI: 2.43–17.19; *p* = 0.0002; Fig. 4c).

Follow-up data were available for 120 individuals. We used Kaplan–Meier analysis with a log-rank test to determine PFS in patients from all groups. Patients with prostate cancers with high *∆133TP53β* (Group A) had a substantially shorter median PFS than Group C (Group A vs C HR = 3.758, 95% CI: 1.59–8.92; *p* = 0.0058; Fig. 4d). Group A also had a shorter PFS than Group B, but this was less dramatic (HR = 1.518, 95% CI: 0.74–3.11; p = 0.049). The survival curves however show very different patterns. PFS of Group A patients begins to decline about 2–4 years after treatment and continues to decline steadily thereafter, whereas Group B patients survive >9 years before there is a decline in survival (Fig. 4d). This suggests that Δ133p53β is driving a more aggressive form of prostate cancer.

To test whether *Δ133TP53β* mRNA levels have predictive value for prognosis, alone or in combination with immune cell infiltration data, *TP53α, TP53β* mRNA levels, T-cell counts, macrophage counts, Gleason score, total prostate specific antigen (PSA) and the University of California, San Francisco (UCSF) Cancer of the Prostate Risk Assessment (CAPRA) score^26^ were assessed for their ability to discriminate high- and low-risk patients by calculating a 10-fold cross-validated area under the curve (AUC). Results (Fig. 4e) show that *TP53β* expression alone can predict high-risk patients with 88% accuracy. By contrast, common biomarkers, PSA levels and Gleason and CAPRA scores had much lower predictive abilities (Fig. 4e).

### RNA sequencing shows enrichment for immune and invasive genes in cancers with elevated *Δ133TP53β* expression

**Fig. 5:**
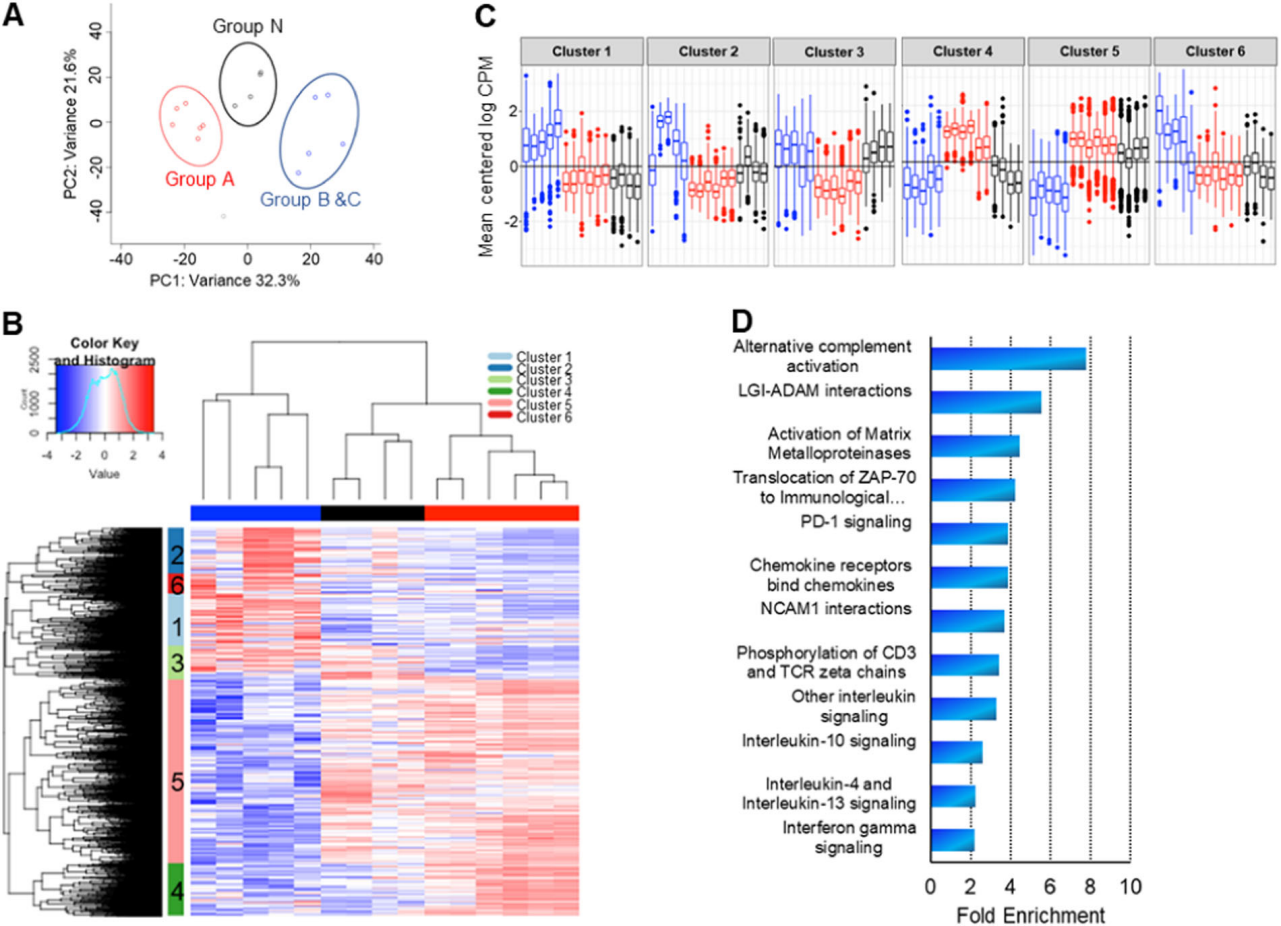
Prostate cancers with elevated *Δ133TP53β* expression show immune and invasive gene signatures. **a** Principal component analyses of 1000 genes with maximum variance determined by RNA-seq of 12 prostate cancers and 4 normal associated tissues. Red—Group A cancers, Blue—Group B and C cancers, and Black—Group N (normal associated tissue). **b** Unsupervised clustering of differentially expressed genes (log fold change ≥1.0 and ≤–1.0 and FDR < 0.05) between Groups N, A and B/C. The vertical bar shows the clusters of genes either up or downregulated across the different groups. Columns—Sample, Row—gene. **c** Shows the mean centred log CPM (log2-counts per million) of genes in each cluster identified by unsupervised hierarchical clustering. Group A – Red, Group B/C—Blue and Group N—Black. **d** Shows fold enrichment of immune and invasive pathways in clusters 4 and 5 using Pantherdb

To provide molecular insight as to how Δ133p53 contributes to prostate cancer progression, we carried out RNA sequencing (RNA-seq) on 12 prostate cancer samples and 4 normal adjacent tissue samples. Principal component analyses (PCA) were done using 1000 genes with maximum variance which identified 3 clusters. Cluster 1 contained 6/12 cancer samples all of which were from Group A, cluster 2 contained 5/12 cancer samples from Groups B and C and cluster 3 comprised 4/4 normal prostate samples (Group N) (Fig. 5a). One cancer sample did not fall into any of these clusters (Fig. 5a). We identified 5420 genes that were differentially expressed among the groups to be significantly altered (FDR < 0.05) by a log fold change of ≥1.0 and ≤–1.0. Unsupervised hierarchical clustering was performed which defined six gene set clusters (Fig. 5b). Clusters 1, 2 and 6 were upregulated in Groups B/C cancers and downregulated in Group A, cluster 3 was upregulated in Group B/C and N and downregulated in Group A. In contrast, cluster 5 was upregulated in Group A and N and cluster 4 was upregulated only in Group A (Fig. 5c). To identify biological processes that are enriched in the different cancer groups, we performed an over-representation test using PantherdB^27^ on all clusters. Clusters 1, 3 and 6 showed no particular enrichment, cluster 2 was enriched for genes involved in lipid metabolism and clusters 4 and 5 were enriched for genes involved in immune regulation, including interferon (IFN)-γ, PD-1 signalling and cell invasion (Fig. 5d).

### Prostate cancers with elevated *Δ133TP53β* have an immunosuppressive infiltrate

**Fig. 6:**
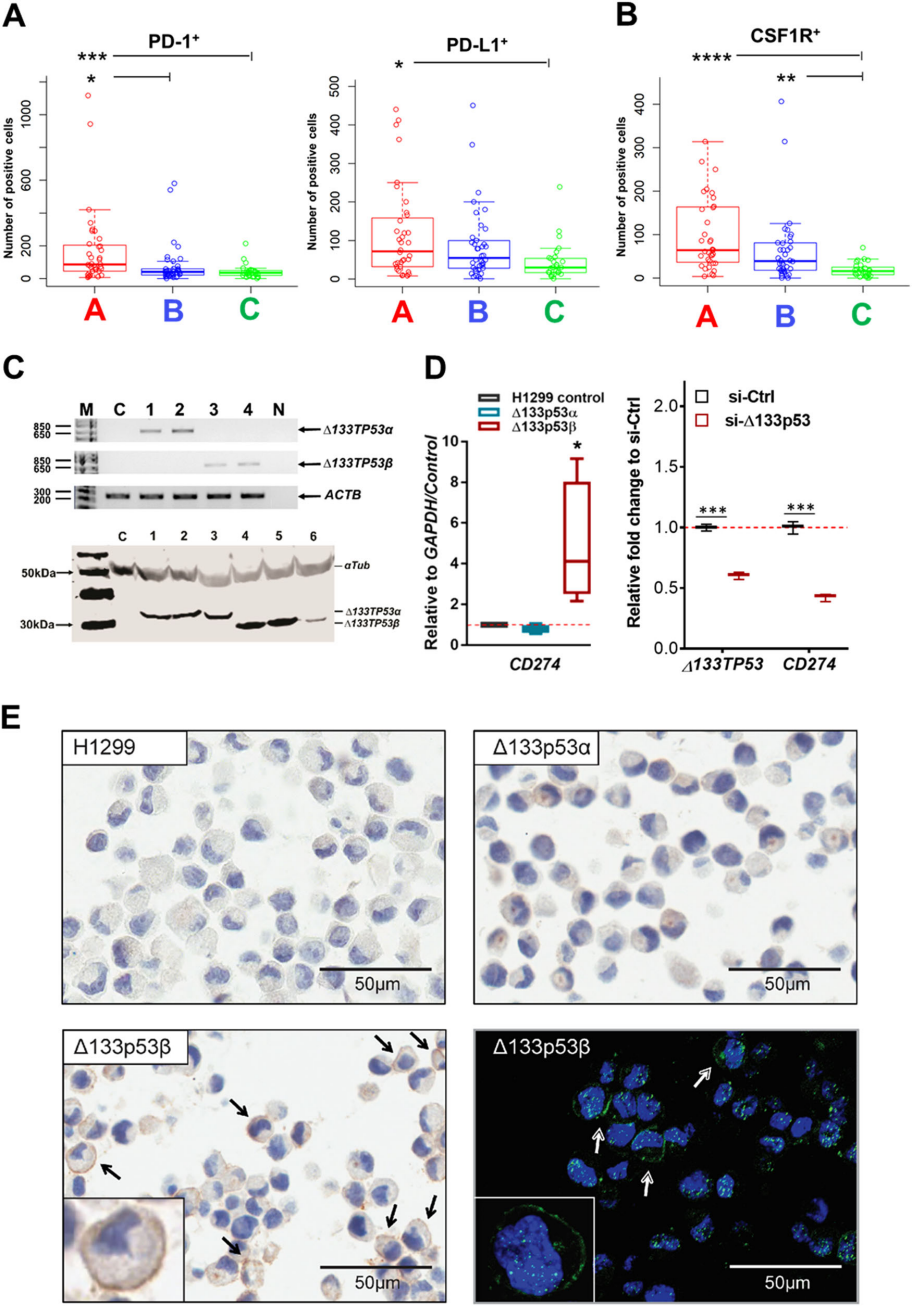
Prostate cancers with elevated Δ133p53β have an immunosuppressive phenotype. **a** Evaluation of an immunosuppressive phenotype by immunohistochemistry shows the number of PD-1 positive (left panel), PD-L1 positive (right panel), and **b** CSF1R positive cells in the three prostate cancer groups. Symbols show individual samples, box (median ± 25th–75th percentile), and whiskers show the 10–90% CI. *p < 0.05, **p < 0.01, ***p < 0.001 and ****p < 0.0001 as determined by Kruskal–Wallis and Dunn’s post hoc test. **c** Top panel: representative nested PCR analysis to validate the expression of *∆133TP53α* (lanes 1 and 2), and *∆133TP53β* (lanes 3 and 4) isoforms in stably transfected p53-null H1299 cells (C control), N non-template control, M molecular weight marker and *ACTB* as a reference gene. Bottom panel: western blot analysis to validate the expression of Δ133p53 isoforms in clonal cells stably transfected with Δ133p53α (lanes: 1–3) and Δ133p53β (lanes: 4–6); the absence of p53 isoforms in p53-null H1299 control cells is also shown (lane: C) and αTub (alpha tubulin) was used as a loading control. **d** Left panel: *CD274*/PD-L1 expression in four clonal lines expressing either Δ133p53α or Δ133p53β isoforms relative to control p53-null H1299 cells. Right panel: *CD274*/PD-L1 expression in 22Rv1 cells 48 h after knockdown of Δ133p53. Box (median ± 25th–75th percentile), and whiskers show the 10–90% CI. *p < 0.05, **p < 0.01, ***p < 0.005 as determined by paired one-tailed *t*-test. **e** Immunohistochemistry and immunofluorescence staining to detect PD-L1 expression in control H1299, Δ133p53α or Δ133p53β expressing cells

Increased immune checkpoint molecules, PD-1 and its ligand, programmed cell death-ligand 1 or 2 (PD-L1 or PD-L2) negatively regulate T-cell-mediated anti-cancer immunity^28,29^. As RNA-seq analysis showed enrichment for PD-1 signalling in Group A cancers, we used IHC to quantitate the number of PD-L1 positive cancer cells and the number of PD-1-positive T cells within each cancer group. Increased numbers of PD-1-positive T-cells were found in Group A cancers (median = 86; 95% CI: 59–173) compared to Groups B (41; 25–53; *p* = 0.013) and C (36; 20–49; *p* = 0.0004; Fig. 6a). The PD-L1 positive cells were also significantly higher in Group A (median = 72; 95% CI: 42–120) compared to Groups B and C (30; 95% CI 17–53; *p* = 0.01; Fig. 6a).

High numbers of tumour-infiltrating macrophages are reported to correlate with poor prognosis for prostate and other cancers^30–32^. Colony-stimulating factor (CSF)-1 controls the differentiation, proliferation, and survival of macrophages by binding to its receptor (CSF1R), expressed on macrophages^33^. Consequently, we measured the number of CSF1R positive macrophages in the prostate cancers. We found Group A and Group B cancers had a higher number of CSF1R-positive cells (median = 65; 95% CI: 51–128 and median = 39; 95% CI: 21–65, respectively) than Group C (16; 8–23; *p* < 0.0001 and *p* = 0.0036, respectively; Fig. 6b).

In summary, prostate cancers with increased *Δ133TP53β* mRNA were characterized by an immunosuppressive phenotype as illustrated by an increased frequency of PD-1, PD-L1 and CSF1R positive cells.

### Δ133p53β directly increases expression of the PD-L1 immune checkpoint marker

To test whether Δ133p53 isoforms could directly increase expression of PD-L1, we created stable transfectants expressing Δ133p53α or Δ133p53β in H1299 p53-null cells. Examples of single-cell-derived clones showing mRNA expression of each isoform are shown in the upper panel and protein expression in the lower panel (Fig. 6c). *CD274* mRNA encoding PD-L1, was quantitated for several isoform clones using RT–qPCR. Results (Fig. 6d) showed that the Δ133p53β expressing cell clones had 2–9-fold (median 4-fold) higher *CD274* mRNA levels than the Δ133p53α expressing and control H1299 cells. These cell clones were also stained for PD-L1 by IHC and immunofluorescence (IF). Increased surface expression of PD-L1 was observed only in the Δ133p53β expressing cell clones (Fig. 6e, Supplementary Fig. S6).

To confirm that Δ133p53β can regulate *CD274* expression in prostate cancer, *CD274* mRNA expression was quantitated in PC3 p53 null cells transiently transfected with a Δ133p53β plasmid, or Δ133p53 levels reduced with an siRNA in 22Rv1 prostate cancer cells. Over-expression of Δ133p53β (Supplementary Fig. S7A) increased *CD274* mRNA levels (Supplementary Fig. S7B) and knock down of Δ133p53 reduced *CD274* mRNA (Fig. 6d, right-hand panel). Thus, Δ133p53β can directly regulate *CD274* expression.

### Δ133p53β directly increases expression of genes involved in immune signalling and migration

**Fig. 7:**
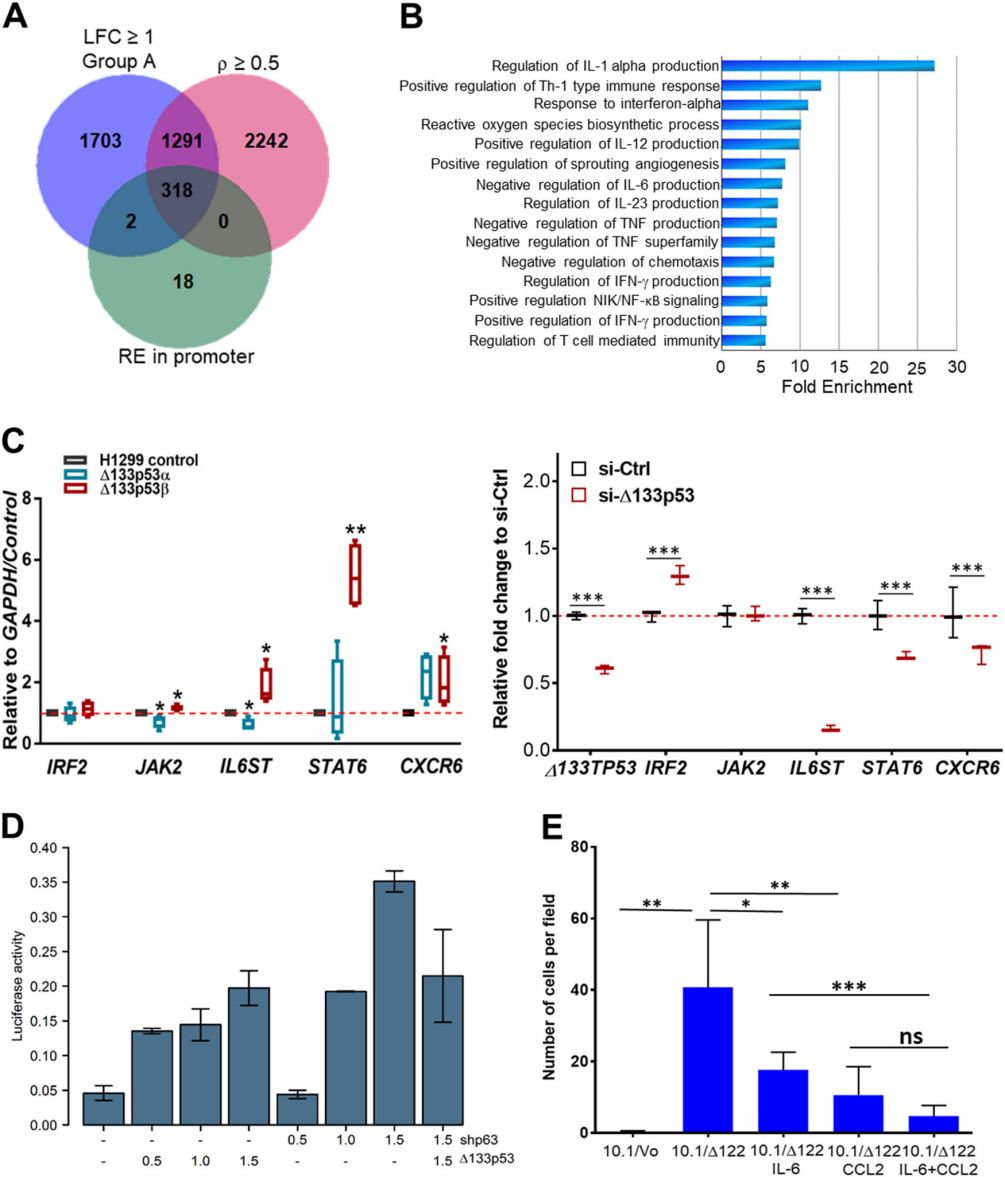
∆133p53 isoforms regulate genes involved in immune cell activity and recruitment. **a** Venn diagram showing genes differentially regulated in Group A prostate cancers, containing p53/p63/p73 response elements in their promoters and are associated with *Δ133TP53β* mRNA expression (Spearman's correlation coefficient cutoff of *ρ* > 0.5). **b** Bar graph depicting a selected list of pathways (PantherdB) with >5 fold enrichment and FDR < 0.05. **c** Expression of selected genes in left panel: four clonal lines expressing Δ133p53α or Δ133p53β isoforms compared to control p53-null H1299 cells, right panel: 22Rv1 cells 48 h after knockdown of Δ133p53. Box (median ± 25th–75th percentile), and whiskers show the 10–90% CI. *p < 0.05, **p < 0.01 and ***p < 0.001, as determined by paired one-tailed *t*-test. **d** Transcriptional activation of the *IL-6* promoter by Δ133p53 or by inhibiting *TP63* (shp63). Cells were transiently transfected with 1.0 µg of IL-6 luciferase reporter plasmid and varying amounts of either Δ133p53 or shp63. Luciferase activity was determined and is normalized to cell number. Bars represent the mean and error bars are ± SD; *n* = 4 biological replicates. **e** 10.1/vector and 10.1/Δ122 cells treated with blocking antibody against either IL-6, CCL2 or both IL-6 (2.0 μg) and CCL2 (3.0 μg). Cells were allowed to migrate for 4 h then membranes were fixed, stained, imaged and quantified. Three technical replicate counts of cells per field were combined and are shown as mean ± SEM. Significance was determined as *p < 0.05, **p < 0.01, ***p < 0.005 using unpaired *t*-tests. ns not significant

The above data indicate that Δ133p53β can increase transcription of multiple genes. However, as Δ133p53 isoforms lack the transactivation domain of p53 and part of the DNA binding domain, to regulate gene transcription it seems likely that the isoforms require one or more co-factors. One of these could be p63 as shown in recent reports^34,35^. To see if any of the above genes/pathways could be regulated by Δ133p53α or Δ133p53β, we identified 318 genes defining Group A cancers that had p53/p63/p73 response elements in their promoters^36^ (Spearman’s correlation coefficient (*ρ*) cutoff of >0.5, Fig. 7a). Analysis of these 318 genes using Pantherdb again enriched for those involved in immune regulation including genes involved in the IFN-γ and NFκB signalling pathways (Fig. 7b). To investigate whether any genes in these pathways are directly regulated by Δ133p53 isoforms, we again used the stable cell lines. We quantitated the transcript levels of genes involved in the IFN-γ response [interferon regulatory factor 2 (*IRF2*), Janus Kinase 2 (*JAK2*); IL-6 receptor subunit beta (*IL6ST*); *STAT6* and C-X-C chemokine receptor type 6 (*CXCR6*)] that had previously been shown to be regulated by p63 and/or one of the Δ133p53 family members^34^. Results show that Δ133p53β expressing H1299 cell clones had elevated *JAK2, STAT6* and *IL6ST* mRNA levels compared to control cells and Δ133p53α and Δ133p53β cell clones had elevated *CXCR6* levels (Fig. 7c). In PC3 cells transiently expressing Δ133p53β (Supplementary Fig. S7A), there was a significant increase in the expression of *STAT6* and *CXCR6* (Supplementary Fig. S7B) and knockdown of Δ133p53 in 22Rv1 cells resulted in a significant reduction in *IL6ST*, *STAT6* and *CXCR6* expression (Fig. 7c). Also in transient transfection experiments, neither wild type (WT) nor mutant p53R175H increased expression of these genes (Supplementary Fig. S7E). These results are consistent with Δ133p53-dependent transactivation utilizing a p53/p63/p73 response element and therefore involving an interaction with p63 as hypothesized^35^.

To test this further, we investigated whether *IL-6* transcription was increased by the isoforms, as we had previously shown that the Δ122p53 mouse mutant had increased levels of IL-6 in serum^9,14,37^. Cells were transfected with Δ133p53α along with an IL-6/luciferase reporter and in parallel with a short-hairpin to *TP63*. Results showed that Δ133p53α increased *IL-6* promoter activity in a dose dependent manner as did reducing *TP63* (Fig. 7d). In combination they were not synergistic, suggesting they work in concert. Again, this is consistent with Δ133p53 interacting with p63 to regulate transcription.

IL-6 contributes to immune cell recruitment and to cancer cell metastasis^37^. Also, CCL2 which is required for macrophage migration^38^ is increased in Δ122p53 expressing cells^14,18^. To test the importance of these molecules in promoting cell migration aided by Δ122p53, p53 null mouse cells transduced with a retrovirus expressing Δ122p53 were used in transwell assays. Results (Fig. 7e) show that cells expressing Δ122p53 migrate about 40-fold more than control cells, which was reduced in the presence of neutralizing antibodies to IL-6 (2-fold) and CCL2 (4-fold); and in combination, 8-fold.

In summary, collectively these data show that Δ133p53 isoforms can regulate genes involved in immune signalling that have a direct bearing on immune cell activity and recruitment.

### Hypoxia stimulates *Δ133TP53* gene expression in prostate cancer cells

Hypoxia is a common characteristic of prostate cancers and plays a key role in prostate cancer growth, aggressiveness and progression^39^. We recently showed that hypoxia contributes to *Δ133TP53β* upregulation in glioblastoma^18^. To determine whether hypoxia could be responsible for elevated *Δ133TP53β* in prostate cancers^8^, we carried out in vitro experiments.

**Fig. 8:**
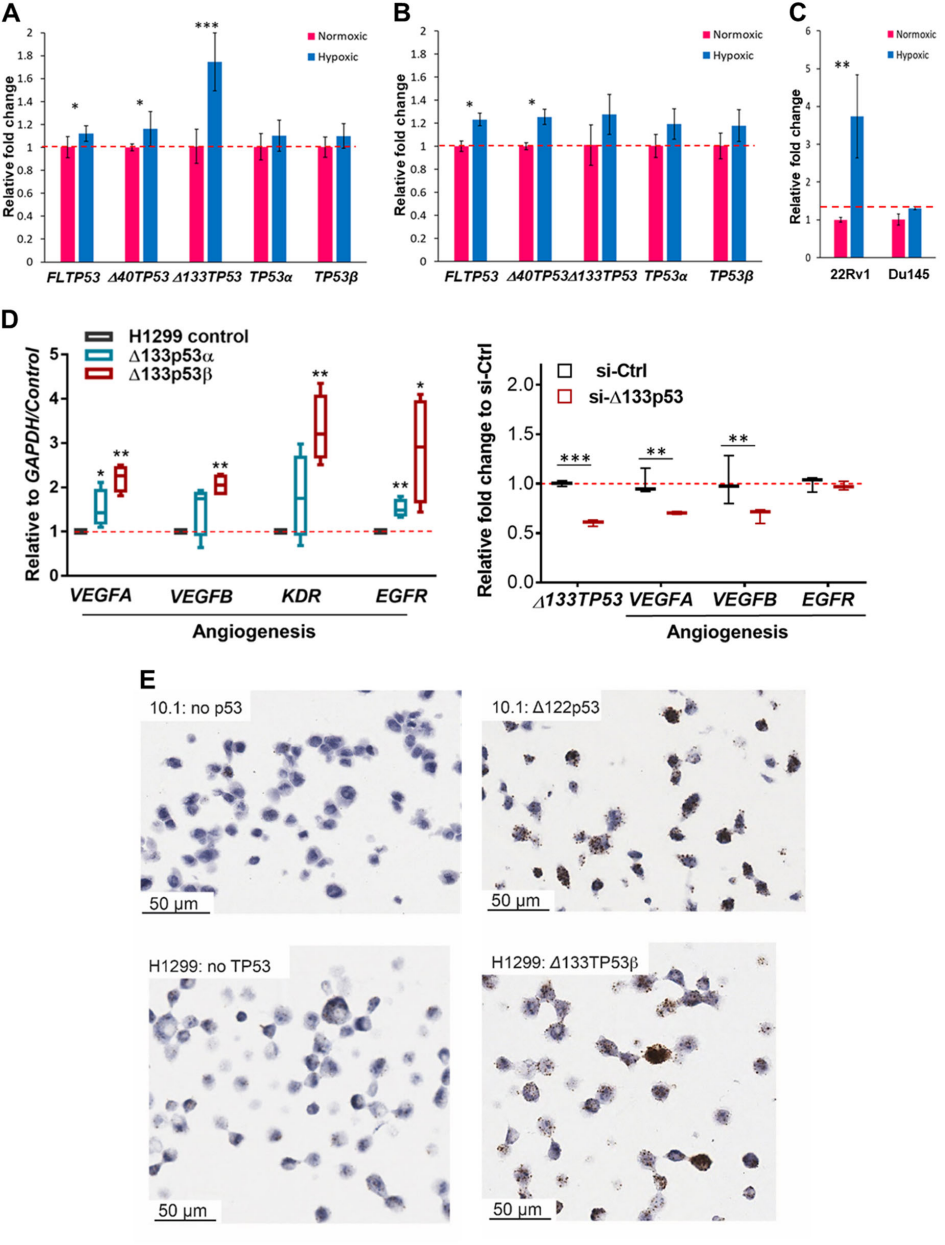
Hypoxia induces expression of the *Δ133TP53* isoform in prostate cancer cells with wild-type p53. Bar plots show relative *TP53* variant expression in **a** 22Rv1 and **b** DU145 cells and in **c**. *VEGFA* expression in prostate cancer cell lines cultured under hypoxic conditions (1% O_2_) for 24 h (blue boxes) compared to those cultured under normoxic conditions (red boxes). *p < 0.05 and ***p < 0.001 as determined by paired one-tailed *t*-test. **d** Expression of selected genes involved in angiogenesis in: left panel: four clonal lines expressing either Δ133p53α or Δ133p53β isoforms compared to control p53-null H1299 cells, right panel: 22Rv1 cells 48 h after knockdown of Δ133p53. Box (median ± 25th–75th percentile), and whiskers show the 10–90% CI. *p < 0.05, **p < 0.01 and ***p < 0.001, as determined by paired one-tailed *t*-test. **e** In situ hybridization using RNAscope to detect *VEGFA* mRNA. Top panel: the mouse p53-null fibroblast cell line 10.1 transduced with a retrovirus expressing Δ122p53 or the control vector. Bottom panel: control H1299 and Δ133p53β expressing cells

Two prostate cell lines, 22Rv1 (WT p53 DNA binding domain (DBD), but has a heterozygous mutation at the C terminus WT/Q331R, which affects the *TP53β* splice) and DU145 (compound heterozygous mutant in the DBD, P223L/V274F)^40,41^ were selected for hypoxia experiments. Cells cultured in 1% O_2_ for 24 h showed an increase in *TP53* transcripts compared to cells in normoxia. In 22RV1 cells *Δ133TP53* mRNA was elevated 1.7-fold whereas there was <10% increase in *FLTP53* and *Δ40TP53* mRNAs (Fig. 8a). We were unable to detect Δ133p53 protein due to very low abundance. In the DU145 cells, hypoxia did not significantly increase *Δ133TP53β* mRNA whereas hypoxia caused a small increase in *FLTP53* and *Δ40TP53* mRNAs (Fig. 8b). Experiments using lung cancer (A549: WT p53) and breast cancer (MDA-MB-231: homozygous mutant p53 DBD, R280K) cell lines showed hypoxia led to induction of *Δ133TP53* and *3’TP53β* expression in A549 cells but not MDA-MB-231 cells (Supplementary Fig. S8). Vascular Endothelial Growth Factor A (*VEGFA*) expression was also measured to confirm the hypoxic environment. This was increased about 4-fold in 22RV1 and A549 cells but was not altered in DU145 or MDA-MB231 cells (Fig. 8c; Supplementary Fig. S8) suggesting WT p53 enhances a hypoxic environment.

To confirm that the isoforms can directly regulate genes associated with hypoxia, we again used the clonal lines. Results (Fig. 8d) show that Δ133p53α and Δ133p53β both increased expression of *VEGFA* and the gene encoding Epidermal Growth Factor Receptor (*EGFR*), whereas only Δ133p53β significantly increased expression of *VEGFB* and the Vascular Endothelial Growth Factor Receptor 2 (*VGFBR2*/*KDR*) gene. PC3 cells transiently expressing Δ133p53β (Supplementary Fig. S7A) also resulted in a significant increase in *VEGFB* expression (Supplementary Fig. S7B) and knockdown of Δ133p53 in 22Rv1 cells resulted in significant reduction in *VEGFA* and *VEGFB* expression (Fig. 8d).

As controls, cells transiently transfected with WT p53 or p53R175H mutant failed to alter expression of these genes, with the exception that WT p53 increased KDR expression (*p* = 0.0015; Supplementary Fig. S7) and its well-known target gene *CDKN1A*. Increased expression of *VEGFA* was also found using RNAscope on the Δ133p53β expressing cell lines and in mouse cells expressing the Δ122p53 mutant (Fig. 8e; Supplementary Fig. S9 for semi-quantitation).

## Discussion

This study investigated an association between expression of *TP53* isoforms, inflammation and prostate cancer progression. We found that cancers with high Δ*133TP53β* mRNA (Group A) were associated with (1) a high proliferative index; increased (2) CD3+ T cells, (3) PD-1 on infiltrating T-cells, (4) PD-L1 on cancer cells, (5) CD163 and CSF1R-positive macrophages; and poor patient outcome. However, we cannot exclude some contribution of the Δ160p53β isoform, which begins translation at an internal initiation codon within *∆133TP53β*^7^. In addition, using multivariate AUC modelling, *∆133TP53β* mRNA levels alone predicted poor outcome at 88% accuracy, much higher than any other single or combination of parameters, implying that Δ133p53β is a principal driver of cancer aggressiveness. By contrast, prostate cancers with increased levels of *Δ40TP53* and *TP53α* mRNAs (Group C) were associated with good prognosis. Our findings for Group A cancers are thus consistent with reports that inflammation is a risk factor for aggressive prostate cancers^3,31,42,43^.

Gene enrichment analysis of cancers also showed that Δ133p53β was associated with pathways involved in immune signalling, along with a transcriptional enrichment for PD-1 signalling, invasion (activation of matrix metalloproteinases) and angiogenesis. These pathways were confirmed using prostate cancer and other cells expressing individual isoforms in which several immune signalling genes including *CD274/*PD-L1, and angiogenesis genes, were upregulated. We also showed that *IL-6* is directly regulated by Δ133p53 and that the mouse mutant mimic of Δ133p53 (Δ122p53) promoted cell migration in transwell assays that were inhibited by antibodies to IL-6 and CCL2. Collectively, our data suggest that Δ133p53β regulates expression of genes involved in immune signalling which likely contributes to immune cell recruitment.

How *Δ133TP53β* mRNA is elevated in prostate and other cancers is not entirely clear, but multiple possibilities have been suggested including infection and response to therapeutic agents^44,45^. Here we showed that hypoxia increased *Δ133TP53* mRNA levels in cells with WT p53. Hypoxia-associated genes were also shown to be directly regulated by Δ133p53 isoforms. Thus, chronic hypoxia may contribute to elevation of *Δ133TP53β* mRNA levels^18^.

Taken together, we propose that in a subset of prostate cancers, chronic stress such as hypoxia in the presence of WT p53 provides signals to increase *Δ133TP53* transcription. The isoforms then turn on genes encoding signalling molecules that recruit immunosuppressive CD163+ macrophages and PD-1+ T cells into the cancer. This combined with increased PD-L1 on the tumor creates an immunosuppressive microenvironment conducive to more aggressive cancers developing. In addition, our data show *Δ133TP53β* mRNA level alone is a highly accurate predictive biomarker for aggressive prostate cancers, which identifies patients that may respond to immune checkpoint therapies.

## Materials and methods

### Patients and tissue specimens

Prostate tissues from prostatectomy or biopsy were obtained from 122 men diagnosed with prostate cancer (Supplementary Table S1). Thirty cancers had normal adjacent tissue available. Ethical approval (LRS/10/09/037/AM05 and 16/STH/92) was obtained and all individuals gave written informed consent. All procedures followed institutional guidelines.

### Cell culture, plasmids and transfection

The human prostate cancer cell lines 22Rv1, DU145 and PC3; breast: MDA-MB-231 and MCF7; osteosarcoma: saos-2; and lung cancer: A549 and H1299 cell lines were obtained from ATCC. Cells were incubated in a humidified atmosphere containing 5% CO_2_ at 37 °C and cultured in DMEM supplemented with 10% FBS for MDA-MB-231, MCF7, saos-2 and A549 cells; RPMI with 10% FCS for 22Rv1, DU145 and H1299 cells; and Ham’s F-12K medium supplemented with 10% FBS for PC3 cells.

For experiments in hypoxic conditions, the cells were cultured in a sealed hypoxia incubator for 24 h. The oxygen level in this incubator was maintained at 1% with the residual gas mixture containing 94% nitrogen and 5% carbon dioxide. After hypoxia exposure the media was removed and the cells were frozen in the plates on dry ice and stored at −80 °C until further analysis.

Plasmids pCMV, pCMV/*Δ133TP53α* and pCMV/*Δ133TP53β* were a kind gift from Dr Jean-Christophe Bourdon (Jacqui Wood Cancer Centre, University of Dundee, Dundee, UK). The pCMV-Neo-Bam p53 wild-type^46^ was obtained from Addgene (Cambridge, MA, USA). Plasmids expressing the p53R175H mutant were generated with site-directed mutagenesis using the PfuUltraHF DNA polymerase (Agilent) and specific primers (Supplementary Table S2). The plasmids were transfected into H1299 cells using Lipofectamine 3000 (Invitrogen, Carlsbad, CA, USA). The selection of clones stably expressing each isoform was carried out for 2 weeks with 800 μg/mL G418 selection (Invitrogen, Carlsbad, CA, USA). Monoclonal cell populations were established using single-cell sorting. Confirmation that the correct isoform was produced was determined by previously described nested PCR^15^ and western blotting. The pCMV vector and *Δ133TP53β* plasmids were also transfected into PC3 cells transiently using Lipofectamine 3000 (Invitrogen, Carlsbad, CA, USA).

### siRNA transfection

22Rv1 cells were reverse transfected with 25bp duplex siRNAs targeted to *Δ133TP53* (si-Δ133p53; target site 5′-GUUGCAGGAGGUGCUUACGCAUGUU-3′) and a scrambled control siRNA (si-Ctrl 5′-CCACACGAGUCUUACCAAGUUGCUU-3′)^34^. The control siRNA has no known human mRNA targets and has been used in previous studies^34^. Stealth siRNAs were transfected at a final concentration of 10 nM using Lipofectamine RNAiMax (Invitrogen). Both siRNAs and RNAiMax were diluted in medium without serum. After 10 min at room temperature, the diluted RNAiMax was added to the siRNAs, and the mixture was incubated for a further 15 min. The lipoplexes formed were added to cells. After overnight transfection, the culture medium was replaced with media supplemented with 10% FBS until the cells were harvested at 48 h. All transfections were performed in triplicate.

### Western blotting

Proteins were isolated using lysis buffer (M-PER™ Mammalian Protein Extraction Reagent, ThermoFisher Scientific, Waltham, MA, USA) supplemented with a protease/phosphatase inhibitor cocktail. The lysates (50 μg) were boiled in SDS sample buffer, separated on SDS-polyacrylamide gels and then electroblotted onto a nitrocellulose membrane. Immunoreactive protein bands were detected using the Odyssey Scanning System (LI-COR Inc., Superior St., Lincoln, NE, USA). The following antibodies were used for western blotting: KJCA (specific for Δ133p53α and β isoforms) at a 1 in 400 dilution; DO-11 (all p53 isoforms) at a 1 in 1000 dilution; DO-7 (FLp53) at 1 in 1000 (Cell Marque, Rocklin, CA, USA); and α-tubulin (Cell Signalling, Danvers, Massachusetts, USA), at a 1 in 20,000 dilution followed by a fluorescein isothiocyanate-coupled secondary donkey anti-rabbit antibody and a near infrared donkey anti-mouse (LI-COR Inc., USA), both diluted 1 in 20,000.

### Preparation of RNA, cDNA synthesis and RT–qPCR for analysis of p53 isoforms

Normal human prostate RNA was obtained from Ambion (Austin, TX, USA) and Clontech (Palo Alto, CA, USA). Total RNA was prepared by PureLink™ RNA Mini Kit (Invitrogen, Carlsbad, CA, USA) and reverse-transcribed using the qScript cDNA synthesis system (Quanta Biosciences, Gaithersburg, MD, USA). Real-time quantitative PCR (RT–qPCR) was performed with a LightCycler® 480 System (RochemDiagnostics, Basel, Switzerland) using SYBRGreen Master Mix (TaKaRa Bio, Otsu, Japan). Reactions used 50 ng of cDNA, were run in duplicate, and a mean value of the two samples calculated. Relative expression levels of each gene were quantified by the 2^−ΔCt^ method using glyceraldehyde 3-phosphate dehydrogenase *(GAPDH)* as an endogenous control. A published nested PCR approach was used to verify the presence of the *Δ133TP53* transcripts in control and clonal cells expressing *Δ133TP53* isoforms^15^. The primers used for RT–qPCR are shown in Supplementary Table S2 and primers for *TP53* variants, *GAPDH, CDKN1A, CXCR6, JAK2, IRF2, IL6ST, STAT6* are as previously described^18,34,47^.

### Luciferase reporter analysis

Saos-2 cells were transfected using FuGENE6 (Promega, Fitchburg, WI, USA) with an *IL-6* Luc reporter construct (SwitchGear Genomics, Carlsbald, CA, USA) at 1 µg per well of a 6-well plate (seeded at 2 × 10^5^ cells) along with either an increasing amount of Δ133p53 expression construct or a short-hairpin targeting p63 (sh-p63) (Origene, Rockville, MD, USA). Forty-eight hours post transfection lysates were collected using the Promega Luciferase Assay System and luciferase activity determined according to the manufacturer’s instructions.

### Transwell assays

Cells were serum starved in medium with 0.5% FCS for 24 h while sub-confluent, then harvested, resuspended in serum deficient medium, and seeded into 8 μm Transwell inserts at 1.25 × 10^4^ cells per insert and placed into 24-well companion plates containing DMEM + 10% FCS as a chemoattractant stimulus. After 4 h cells were fixed with 4% paraformaldehyde, stained with 3% crystal violet, and non-migratory cells removed from the inside of the insert with a cotton bud. Membranes were then imaged using an inverted research microscope (Olympus, Tokyo, Japan) with DP71 microscope digital camera (Olympus, Tokyo, Japan) with six images taken per membrane. Each set of images was taken in the same place for each membrane to minimize any bias and ensure consistency. Images were then analysed using ImageJ (Image Processing and Analysis in Java; US National Institutes of Health, Bethesda, MD, USA). Data are presented as the number of cells per field, which represents the mean of the counts across the six fields for each membrane. Replicate measurements (number of cells per field) were then combined and data analysed using unpaired *t*-tests.

### Immunohistochemical and immunofluorescence examination

Four-µm sections from formalin fixed paraffin-embedded tissues were used for IHC and IF. IHC staining was performed for CD3, CD4, CD8, CD20, CD163, CSF1R, PD-1, PD-L1, Ki67, and p53β (KJC8)^6,18^ and IF staining was also performed for PD-L1.

The primary antibodies and criteria for evaluating staining are in Supplementary Table S3. All antibodies with the exception of those detecting CSF1R and p53β were subjected to automated IHC with heat-mediated epitope retrieval, and diaminobenzidine chromogen (DAB) detection reagents (Leica Biosystems, Wetzlar Germany). To detect CSF1R and p53β manual IHC was done. Antibodies were incubated on tissue sections overnight at 4 °C before detection using EnVision Dual Link (Dako, Glostrup, Denmark) and DAB (Cell Marque, Rocklin, CA, USA) with DAB enhancer (Leica Biosystems, Wetzlar, Germany). Antibodies were diluted in Primary Antibody Diluent BOND (Leica Biosystems, Wetzlar, Germany) or Van Gogh Diluent (Biocare Medical, Pacheco, CA, USA) for p53β. To detect PD-L1 expression by IF, manual staining was performed, using heat-mediated epitope retrieval (0.1 M Citrate buffer pH 6.0). Sections were permeabilized in 0.5% TritonX-100/0.1% BSA prior to blocking in 5% normal goat serum, followed by primary antibody incubation overnight (1:200 dilution) at 4 °C and AlexaFluor–conjugated secondary antibody 488 nm (1:1000; Life Technologies, Carlsbad, CA, USA) for 1 h at room temperature. Nuclei were detected with 1 μg/mL Hoechst dye. All slides were examined and imaged using the Zeiss 710 Confocal Laser Scanning Microscope (Zeiss, Oberkochen, Germany). Confocal *Z* stacks were generated and were reconstructed using an *xy* maximum intensity projection using ImageJ software (Image Processing and Analysis in Java; US National Institutes of Health, Bethesda, MD, USA).

For chromogenic-based staining, positive cells were identified using the Aperio Scancope CS digital pathology system (Aperio, Vista, California, USA) or using the DM 2000 microscope, DFC 295 camera and Application Suite software, version 3.5.0, (Leica, Solms, Germany). PD-L1 staining was quantified using the Aperio Membrane Algorithm (Aperio, Vista, California, USA). Slides were evaluated by two blinded examiners.

### RNAscope

A custom probe to the unique region of *∆133TP53* and *TP53β* was made by Advanced Cell Diagnostics (Advanced Cell Diagnostics, Newark, CA, USA). The probe was designed to *Δ133TP53β* reference sequence DQ186651.1 with the probes between nucleotides +97 and +277 unique to *Δ133TP53* isoforms, but excluding the upstream *AluJb* repeat^47^. To increase the amount of sequence available for probe stability, nucleotides +847–1001 were also included. Probes to *VEGFA* were used on human (reference number 423161) and mouse cells (reference 436961, Advanced Cell Diagnostics, Newark, CA, USA).

Formalin fixed paraffin-embedded cell clots and tumors were cut into 5 µm sections. The RNAscope method used the manual assay 2.5 protocol with Protease Plus reagent for protein digestion and the 2.5HD reagent kit brown for detection of the probe according to the manufacturer’s instructions. The assay was optimized using paraffin-embedded cell clots containing MCF7 and *TP53* null Saos-2 cells (Supplementary Fig. S2). Following addition of DAB, DAB enhancer was added (Leica Biosystems, Wetzlar Germany). Positive cells were identified using the Aperio Scancope CS digital pathology system and quantified using the Aperio RNA ISH Algorithm (Aperio, Vista, California, USA). Slides were evaluated by two blinded examiners.

### RNA sequencing and data analyses

RNA was extracted from prostate tumours and normal adjacent tissue and sequenced using Illumina HiSeq 2500 (Otago Genomics Facility, OGF (https://www.otago.ac.nz/genomics/index.html)). Libraries were constructed and sequenced using a TruSeq Stranded mRNA Library Prep kit (Illumina, San Diego, CA, USA; 500 ng input Total RNA) according to the manufacturer's instructions, and an Illumina HiSeq 2500 (2x125 bp; HiSeq SBS v4 High Output run mode).

Sequencing reads were first adapted and quality trimmed (Q20) using fastq-mcf (https://github.com/ExpressionAnalysis/ea-utils). Reads were then mapped against the hg19 human reference genome using HISAT2 version 2.0.5^48^ (https://ccb.jhu.edu/software/hisat2/index.shtml). Read counts were first retrieved by exon if the mapping quality was higher than Phred score of 10 and then summarized by gene using featureCount^49^ version v1.5.3. Read counts were subsequently normalized using the “median ratio” method. Principal component analysis was performed on regularized log counts. All vs all pairwise comparisons were performed and differentially expressed genes were identified using the DESeq2 R package (https://bioconductor.org/packages/release/bioc/html/DESeq2.html) after correcting for multiple tests using Benjamini-Hochberg method with a threshold of 5% for the False Discovery Rate. Genes significantly different (FDR < 5%) with one fold change were clustered based on their normalized expression using hierarchical clustering approach with a complete linkage method (hclust).

Gene set enrichment was performed on clusters of genes having at least one fold change in one pairwise comparison using the PantherdB^27^ to identify significant pathways that are likely to be associated with high levels of *Δ133TP53β* mRNA.

### *TP53* mutation analysis

DNA was extracted from frozen tumours and used in PCR to amplify exons 4–9 of *TP53*. The primer sequences used were those published^50^. Purified PCR products were subjected to Sanger sequencing to identify mutations.

### Statistical analyses

Pairwise comparisons were done with a Mann–Whitney test. Comparisons across groups were done with a Kruskal–Wallis test followed by Dunn’s Multiple Comparison test. The Spearman’s rank correlation analysis was employed to evaluate correlations between the mRNA levels of pairwise genes. Associations between clinical subtypes were evaluated using the chi-square test. Differences between survival curves were tested using the two-sided log-rank test. Statistical analyses were performed using GraphPad Prism software version 6.00 and R statistical software^51^.

Average linkage hierarchical clustering was performed using the rank() and hclust() in R, using batch normalized (https://rdrr.io/bioc/sva/man/ComBat.html) mRNA expression of *TP53* transcripts; immune cell content (CD3, CD20, and CD163 cell counts); Ki67; and Gleason score. Logistic regression was used to evaluate the ability of *∆133TP53β* expression, T-cell and macrophage counts, Gleason score and total PSA to predict patient outcome. To validate multivariate binary logistic regression model, 10-fold cross-validation with the R package ‘cvAUC’ (version 1.1.0) was performed.

## Supplementary information

Figure S1 - S9, Table S1-S3
