# Supplementary material for: The Δ133p53β isoform promotes an immunosuppressive environment leading to aggressive prostate cancer
Source: Cell Death Dis. 2019 Aug 20;10(9):631. doi: 10.1038/s41419-019-1861-1 (PMC6702175; doi:10.1038/s41419-019-1861-1)
Supplement: Supplementary file 1 — Figure S1 - S9, Table S1-S3 [file 41419_2019_1861_MOESM1_ESM.docx]

**Supplementary Information**


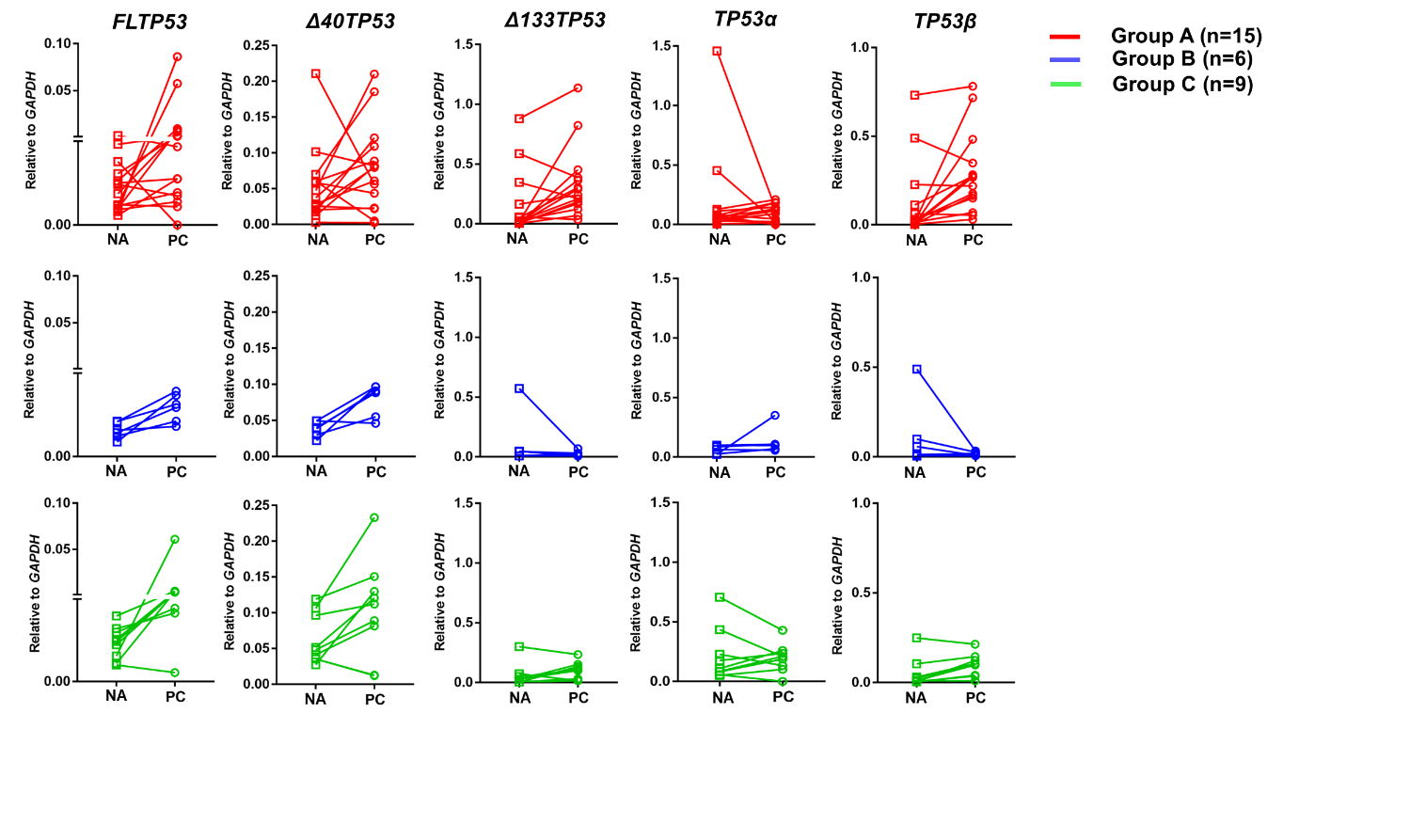


**Figure S1.** *TP53* isoform expression profiles in paired normal and prostate tumor samples.

The *FLTP53*, *Δ40TP53, Δ133TP53*, *TP53α*, *TP53β* expression levels in matched normal adjacent to tumor (NA) and prostate cancer (PC) tissues are represented as relative expression to the reference gene *GAPDH*, using the 2-ΔCt method. Red, blue, and green lines highlight the isoform expression in Groups A, B, and C, respectively.


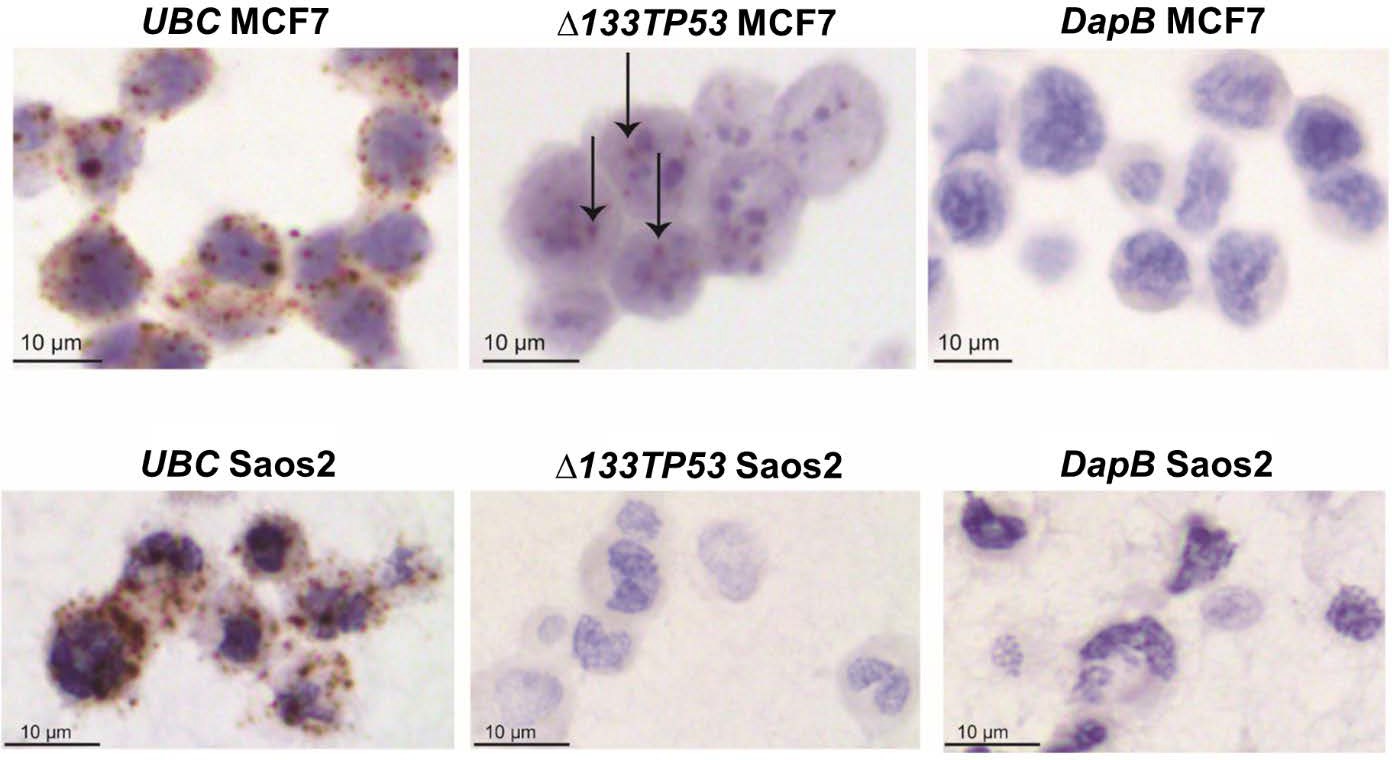


**Figure S2**. Optimization of the *∆133TP53* RNAscope assay using cell lines with and without *∆133TP53*.

Formalin fixed paraffin embedded tissue sections of *TP53* positive (MCF7) and negative (Saos2) cells were subjected to *in situ* hybridization using RNAscope. Top panel MCF7, left *ubiquitin C* (*UBC*) as a positive control for RNA quality, middle panel, probes against *Δ133TP53*, and right panel probes to the bacterial gene *DapB* as a negative control. Bottom panel, Saos2, left *UBC*, middle panel *Δ133TP53*, and right panel *DapB*. Nuclei were counterstained with hematoxylin. Arrows indicate *Δ133TP53* positively stained cells.

**
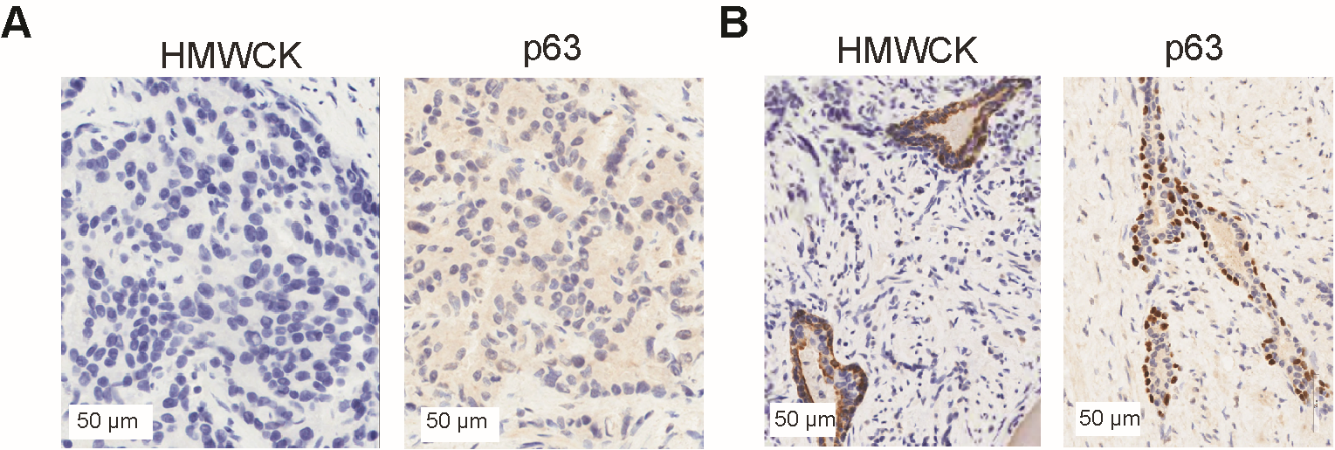
**

**Figure S3.** HMWCK and p63 staining in normal associated tissue but not in prostate cancers.

**A.** Loss of p63 and high molecular weight cytokeratin (HMWCK) staining in prostate cancer. **B.** p63 and HMWCK staining in normal associated tissues.


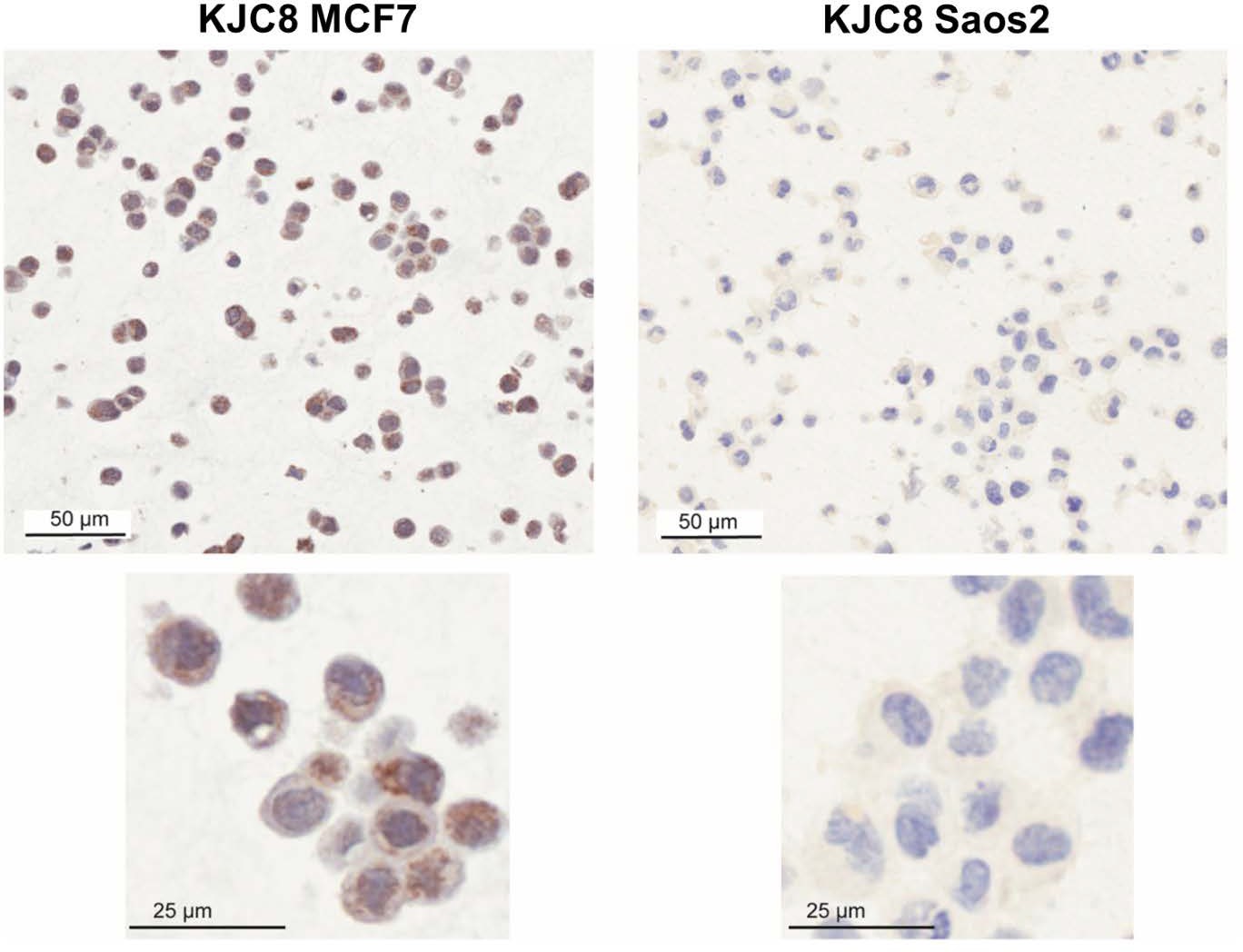


**Figure S4.** Optimization of the p53β immunohistochemistry using cell lines with and without p53β.

Formalin fixed paraffin embedded tissue sections of *TP53* positive (MCF7) and negative (Saos2) cells were subjected to immunohistochemistry using the KJC8 antibody.


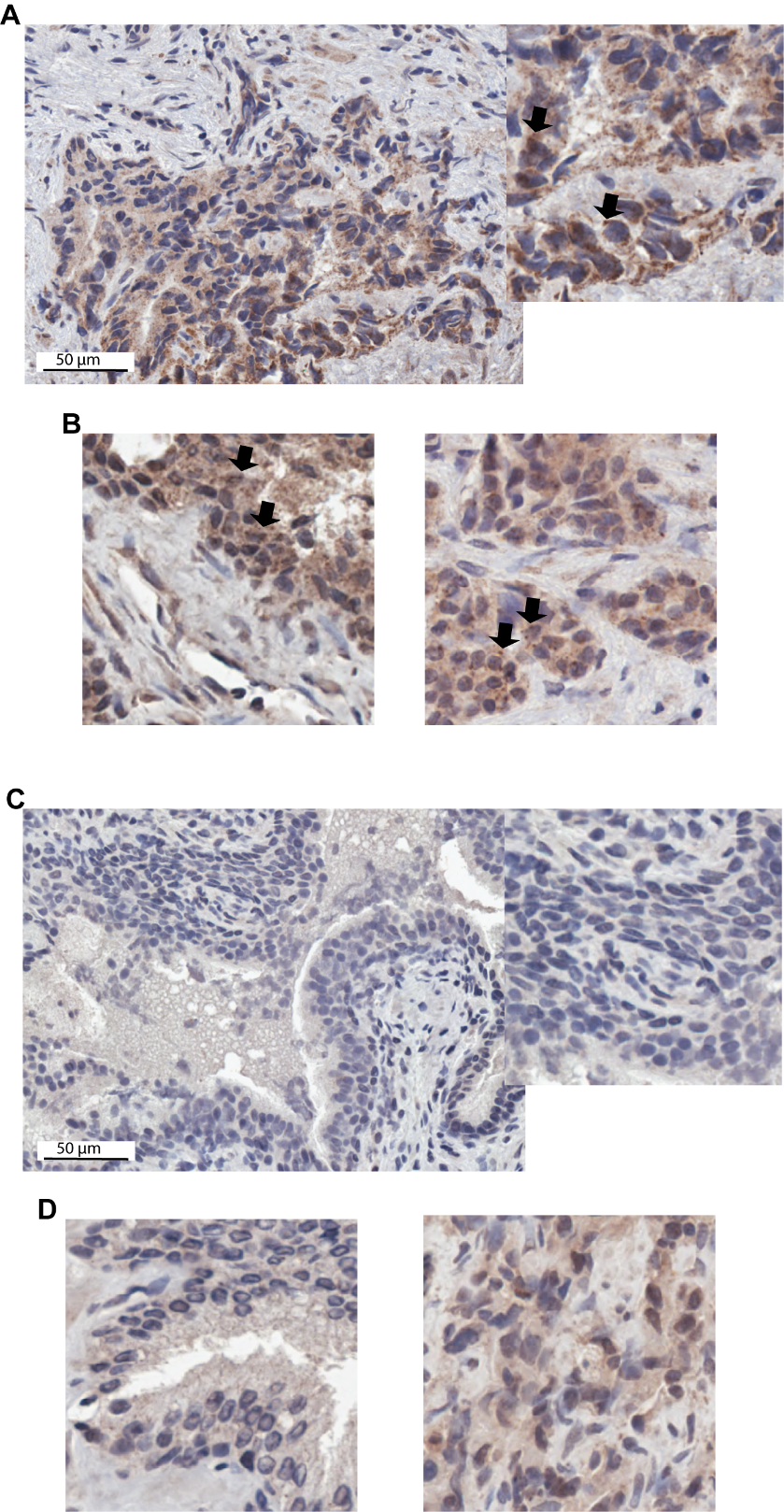


**Figure S5.** p53β is expressed in a subset of prostate cancers.

**A - B.** Example of positive staining for p53β using the KJC8 antibody in FFPE prostate cancer tissues. Black arrows – show positive punctate staining. **C- D.** Examples of no staining for p53β using the KJC8 antibody in FFPE prostate cancer tissues.


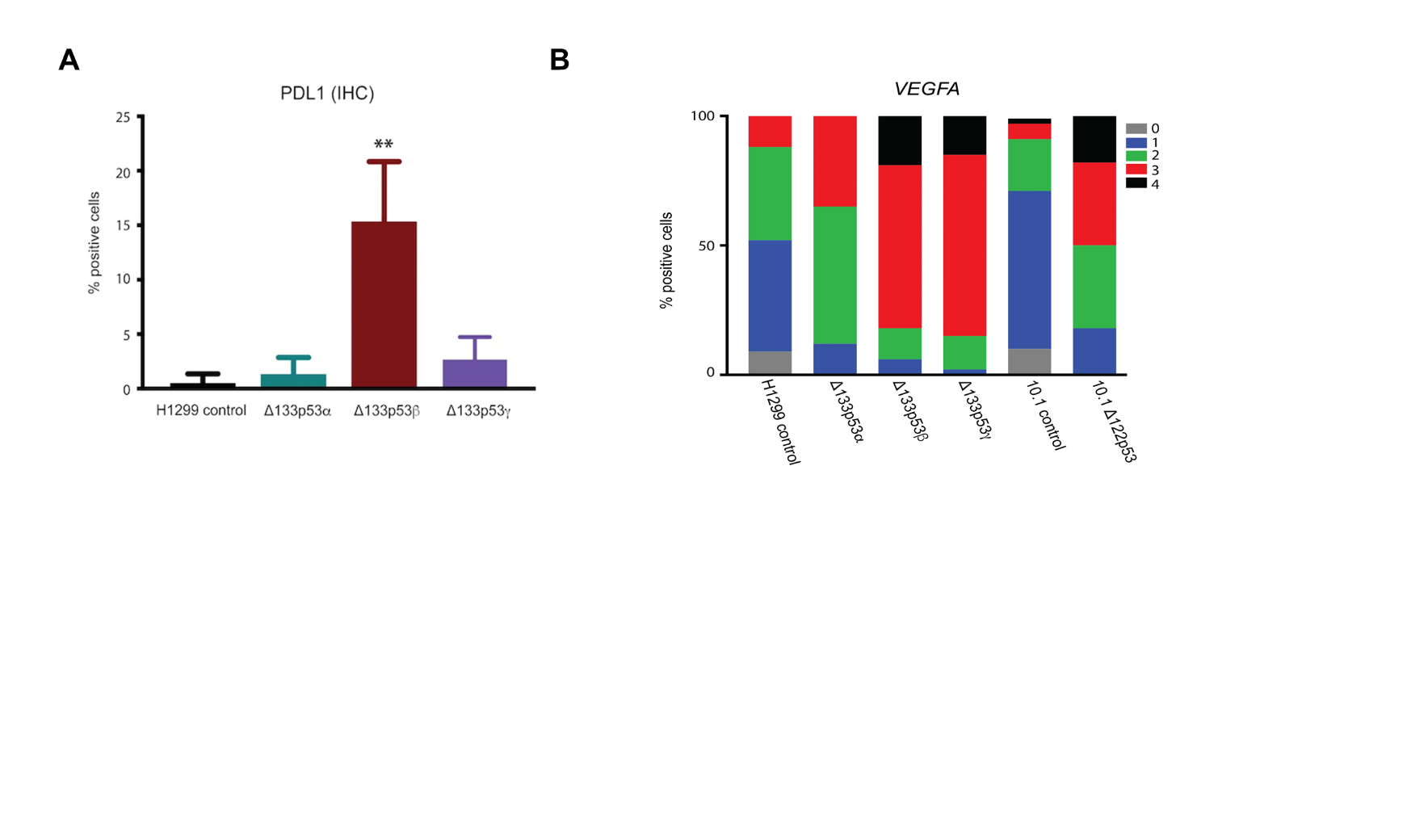


**Figure S6.** Semi quantitation of PD-L1 staining in p53 isoform expressing cells.

**A.** PD-L1 positive cells were determined using immunohistochemistry and compared between H1299 cells stably expressing the Δ133p53 α or β isoform or no isoform (H1299 control). Results are mean ± sd from three separate cultures.


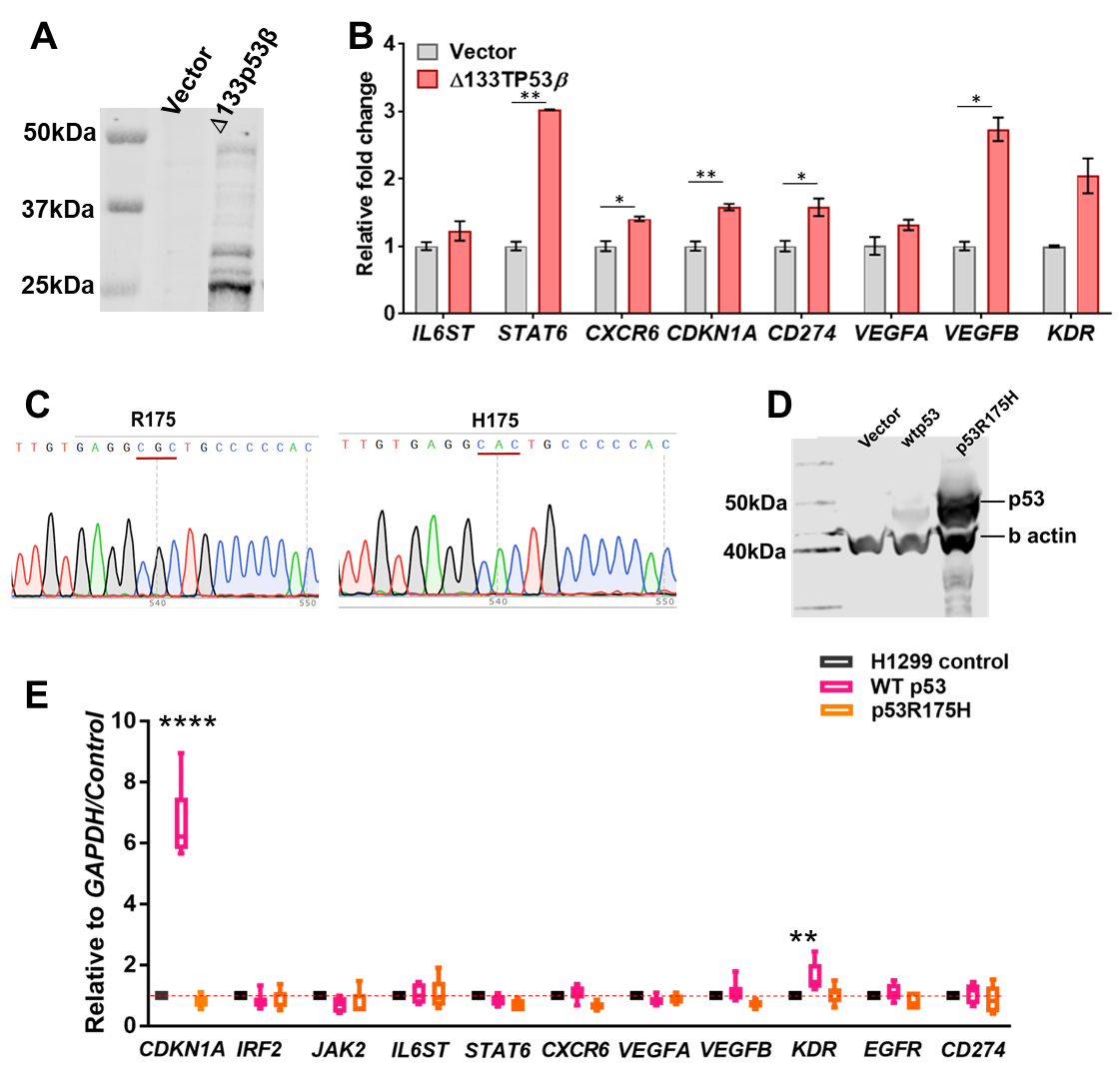


**Figure S7. Neither WT p53 nor p53R175H mutant increased expression of interferon responsive and angiogenesis genes. A.** Western blot confirming expression of Δ133p53β constructs in transiently transfected PC3 cells. **B.** Bar plots showing the levels of expression of selected genes (CDKN1A/p2, IL6ST, STAT6, CXCR6VEGFA, VEGFB, KDR and CD274/PD-L1) in p53-null PC3 cells transiently expressing Vector or Δ133p53β constructs. Bars represent the mean and error bars are ± S.D; n=3 biological replicates. **p<0.01 and ****p<0.0001, as determined by one tailed t-test. **C.** Sanger sequencing chromatograms of: WT p53 (left panel) and p53R175H mutant (right panel). **D.** Western blot confirming expression of WT p53 and p53R175H constructs. **E.** Box and whisker plots show the levels of expression of selected genes (*CDKN1A*/p21, *IRF2*, *JAK2*, *IL6ST*, *STAT6, VEGFA*, *VEGFB*, *KDR*, *EGFR* and *CD274*/PD-L1) in p53-null H1299 cells transiently expressing WT p53 or p53R175H. Box (median ± 25th-75th percentile), and whiskers show the 10 to 90% from n=6 technical replicates. **p<0.01 and ****p<0.0001, as determined by paired one-tailed *t*-test.


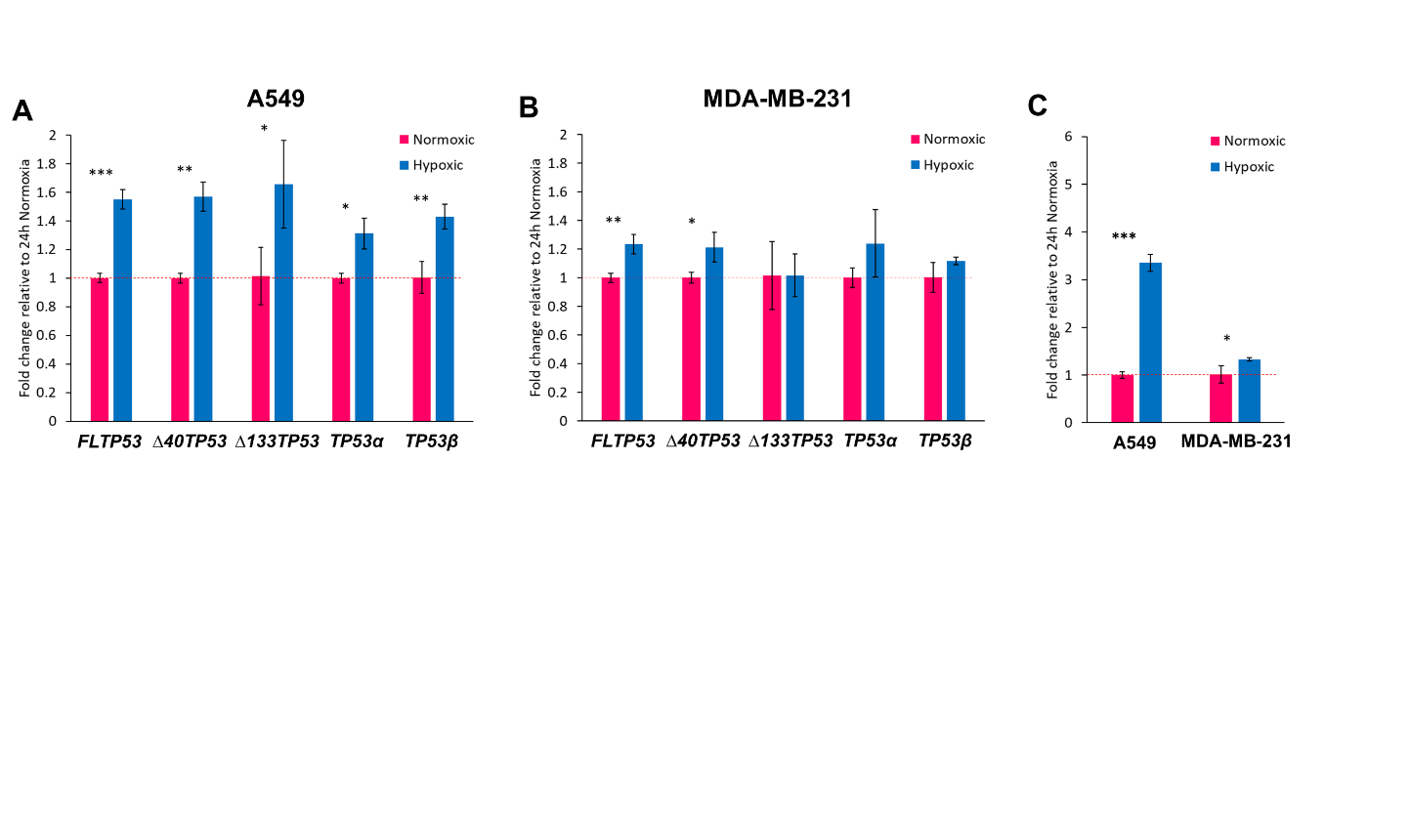


**Figure S8.** Expression of *Δ133TP53* in lung and breast cancer cells with hypoxia.

Bar graphs show relative *TP53* variants expression in **A.** A549 (WT *TP53*) and **B.** MDA-MB231 (mutant *TP53*) and in **C.** Vascular Endothelial Growth Factor A (*VEGFA)* expression in cell lines cultured under hypoxic conditions (1% O_2_) for 24 hours (blue boxes) compared to those cultured in normoxic conditions (red boxes). Bars represent the mean and error bars are ± S.D; n=3 biological replicates. *p<0.05, **p<0.01, ***p<0.001 as determined by paired one-tailed *t*-test.

**Figure S9.** Semi quantitation of *VEGFA* expression in p53 isoform expressing cells.


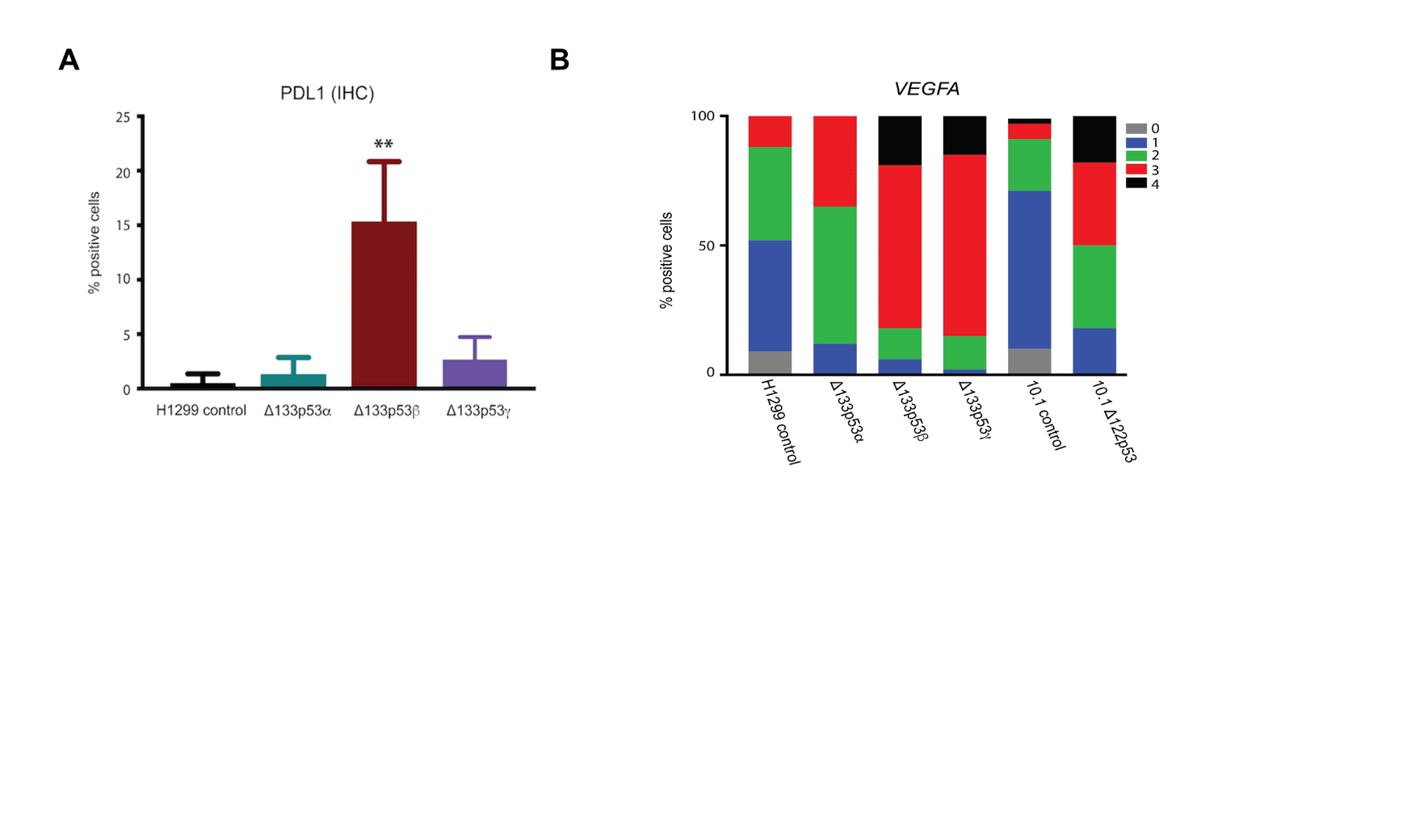

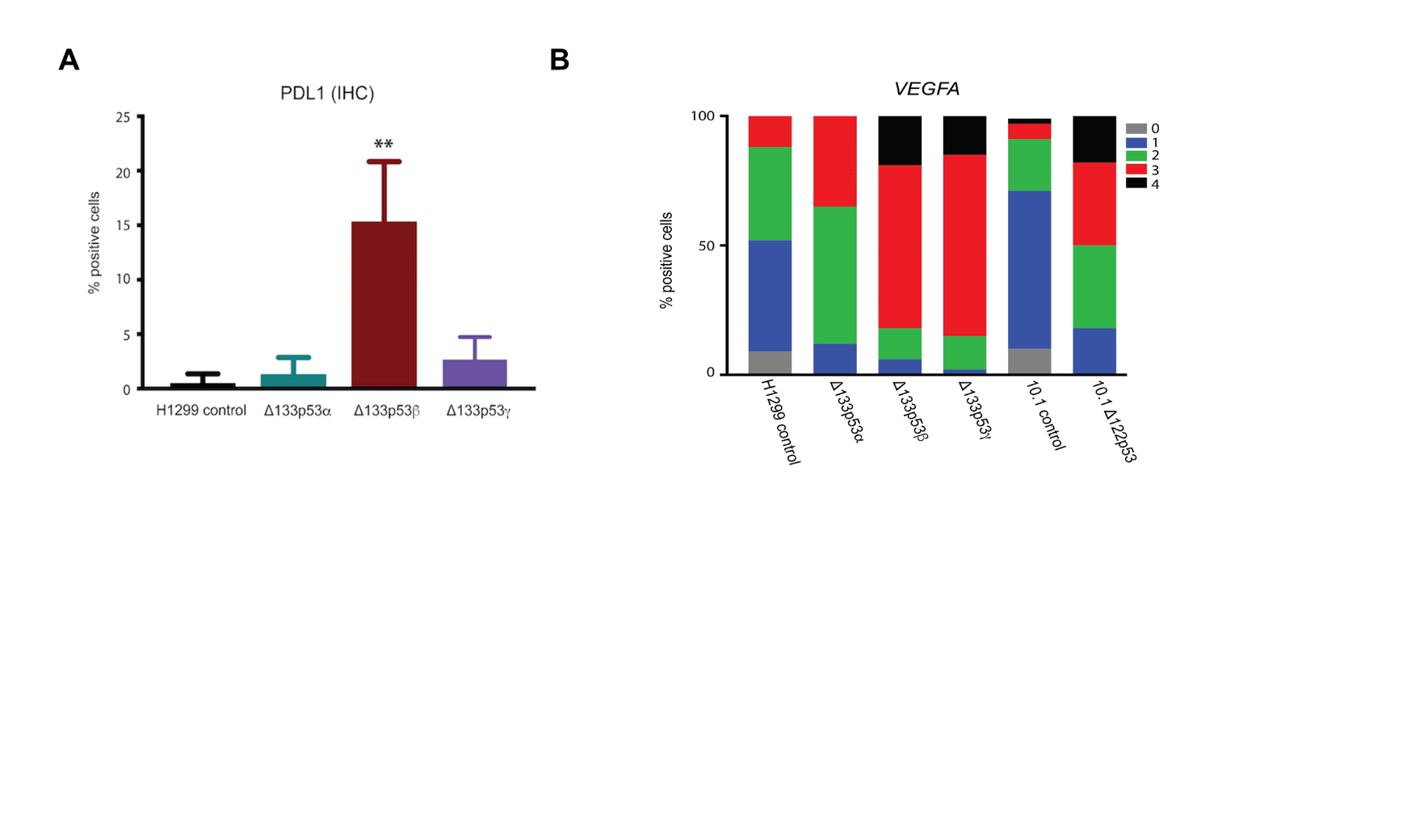


*VEGFA* was measured using RNAscope and the percentage of cells with a specific score system based on the number of dots per cell compared between H1299 cells stably expressing the Δ133p53 α or β isoforms or no isoform (H1299 control) and mouse p53 null 10-1 cells compared with 10-1 cells expressing Δ122p53. Score: 0, no dots per cell; 1, 1-3 dots per cell; 2, 4-9 dots per cells, none or very few clusters; 3, 10-15 dots per cells and <10% dots are in clusters; and 4, >15 dots per cells and >10% dots are in clusters (Advanced Cell Diagnostics, scoring guidelines).

**Table S1.** Demographics and clinical characteristics of patients with prostate cancer in the original and validation cohorts

|  | **Cohort 1** | **Cohort 2** |
| --- | --- | --- |
| **No. of patients** | 43 | 79 |
| **Median age, years (IQR range)** | 63 (59-68) | 66 (60-70) |
| **Median serum total PSA (IQR range)** | 8.75 (7.1-11.5) | 8 (6.2-10.9) |
| **Gleason ≤ 6** | 49% | 38% |
| **7** | 49% | 51% |
| **8-10** | 2% | 11% |

IQR, interquartile range

**Table S2.** Primer sequences used for RT-qPCR and site directed mutagenesis

| **Primer**  **#** | **Gene** | **Sense** | **Primer Sequence (5' - 3')** |
| --- | --- | --- | --- |
| 1 | *CD274* | Forward | GCAGGGCATTCCAGAAAGATG |
| 2 |  | Reverse | AACCGTGACAGTAAATGCGTTC |
| 3 | *EGFR* | Forward | TGAGTGCATACAGTGCCACC |
| 4 |  | Reverse | TACAGTTGTCTGGTCCCCGT |
| 5 | *KDR* | Forward | TGGTTGTGTATGTCCCACCC |
| 6 |  | Reverse | CATGTCAGCGTTTGAGTGGTG |
| 7 | *VEGFA* | Forward | CTTCAAGCCATCCTGTGTG |
| 8 |  | Reverse | GAGGTTTGATCCGCATAATCTG |
| 9 | *VEGFB* | Forward | CCCTTGACTGTGGAGCTCAT |
| 10 |  | Reverse | TGAGGATCTGCATCCGGACTT |
| 11 | *ACTB* | Forward | CATGTACGTTGCTATCCAGGC |
| 12 |  | Reverse | CTCCTTAATGTCACGCACGAT |
| 13 | *p53R175H* | Forward | ATGGTGGGGGCAGTGCCTCACAACCTC |
| 14 |  | Reverse | GAGGTTGTGAGGCACTGCCCCCACCAT |

Primers 1 to 12 were designed for RT-qPCR use. Primers 13 to 14 were designed for site directed mutagenesis.

**Table S3.** Antibody characteristics and criteria for assessment of staining

| **Target** | **Antibody clone** | **Criteria for assessment** |
| --- | --- | --- |
| CD3 | MRQ-39  (Cell Marque, Rocklin, CA, USA) | Amount of cells in 30 hpf |
| CD4 | SP35  (Cell Marque, Rocklin, CA, USA) | Amount of cells in 30 hpf |
| CD8 | SP16  (Cell Marque, Rocklin, CA, USA) | Amount of cells in 30 hpf |
| CD20 | L26 (Dako, Glostrup, Denmark) | Amount of cells in 30 hpf |
| CD163 | 2G12 (Abcam, Cambridge, UK) | Amount of cells in 30 hpf |
| CSF1R | Anti-MCSF receptor, SP211 (Abcam, Cambridge, UK) | Amount of cells in 30 hpf |
| *HMWCK | 34βE12 (Leica Biosystems, Wetzlar Germany) | Used to distinguish malignant prostate tissue |
| Ki67 | MIB-1  (Dako, Glostrup, Denmark) | Amount of positive malignant cells/ total number of malignant cells in 30 hpf*, and expressed as the percentage of positive cells |
| PD-1 | NAT105  (Cell Marque, Rocklin, CA, USA) | Amount of cells in 30 hpf |
| PD-L1 | CAL10 (Biocare Medical, Pacheco,  CA, USA) | Amount of positive in 30 hpf** |
| *P63 | 7JUL (Leica Biosystems, Wetzlar Germany) | Used to distinguish malignant  prostate tissue |
| TP53β | KJC8 (6) | Used in 10 tumors to identify the  cell types positive |

hpf, high power field (x400 magnification), 30 fields were chosen to include as much tissue as possible and was based on the tumor with the least amount of tissue available *, immunostaining for p63 and high-molecular-weight cytokeratin was used to ensure only cancerous areas were examined. **. Total PD-L1 positive cells were counted and included tumor and immune cells. The number of cells or the percentage positive was recorded as the average between the two examiners. HMWCK, high molecular weight cytokeratin.
